# Recent Advancements in the Nanomaterial Application in Concrete and Its Ecological Impact

**DOI:** 10.3390/ma14216387

**Published:** 2021-10-25

**Authors:** Haleema Saleem, Syed Javaid Zaidi, Nasser Abdullah Alnuaimi

**Affiliations:** Center for Advanced Materials (CAM), Qatar University, Doha P.O. Box 2713, Qatar; haleema.saleem@qu.edu.qa (H.S.); anasser@qu.edu.qa (N.A.A.)

**Keywords:** nanomaterials, concrete, nanosilica, risk assessment, health risks

## Abstract

At present, nanotechnology is a significant research area in different countries, owing to its immense ability along with its economic impact. Nanotechnology is the scientific study, development, manufacturing, and processing of structures and materials on a nanoscale level. It has tremendous application in different industries such as construction. This study discusses the various progressive uses of nanomaterials in concrete, as well as their related health risks and environmental impacts. Nanomaterials such as nanosilica, nano-TiO_2_, carbon nanotubes (CNTs), ferric oxides, polycarboxylates, and nanocellulose have the capability to increase the durability of buildings by improving their mechanical and thermal properties. This could cause an indirect reduction in energy usage and total expenses in the concrete industry. However, due to the uncertainties and irregularities in size, shape, and chemical compositions, some nanosized materials might have harmful effects on the environment and human health. Acknowledgement of the possible beneficial impacts and inadvertent dangers of these nanosized materials to the environment will be extremely important when pursuing progress in the upcoming years. This research paper is expected to bring proper attention to the probable effects of construction waste, together with the importance of proper regulations, on the final disposal of the construction waste.

## 1. Introduction

Nanotechnology is a developing field of science related to the engineering of nanosized particles composed of different materials [1,2,3,4,5,6]. Nanotechnology is distinguished as the study of operating matter on a molecular and an atomic scale that permits the conception of innovative techniques and devices with sizes in the range of 1 nm to 100 nm, and the utilization of these materials varies from mechanics to medical applications. This technology generates vast commercial potential for industries and is normally considered as a very promising one. Materials perform in a different manner at the nanoscale level [7], and with the application of nanomaterials, exceptional results can be obtained in different fields [8,9].

In the past several decades, nanotechnology has accomplished incredible development [10,11,12,13,14]. More enhanced awareness of nanomaterial characteristics contributes to finding a better way to fabricate advanced materials in the forthcoming years and can improve standards of living. The development of and research on nanotechnology are extremely active worldwide, and this technology is already being employed in different applications and seen in hundreds of products currently, such as textiles, cosmetics, sports equipment, paint, and electronics [15,16,17,18]. This advanced technology is also being utilized in biosensors, drug delivery, other biomedical uses, and environmental applications (environmental contaminant clean-up) [19,20,21,22]. In recent times, nanotechnology was also found to have tremendous applications in construction industries [23,24,25,26,27,28,29]. Nanomaterials have uses in coatings, glass, concrete, steel, bricks, and insulation.

The chemistry and physics of nanosized construction materials vary from ordinary materials because of quantic effects and a greater surface-area-to-volume ratio. Characteristic morphologies of nanomaterials are sheets, cylinders, and spheres. Currently, the utilization of nanomaterials in the building and construction materials industries has highlighted several benefits [30,31,32,33,34]. In the past few years, significant and multiple progressions in nanoscience and technology have shifted the construction and building material technology to a very advanced level. Innovative trends and opportunities are developing, which must be taken into the account by young researchers, engineers, and companies. Different types of manufactured nanomaterials can have advantageous utilization in construction, including high-resolution sensing/actuating devices, superior structural properties, and functional coatings and paints [35,36,37,38]. Some utilizations of nanomaterials in the construction industry are presented in Table 1 [39,40,41,42,43,44,45].

Concrete is one of the prominent materials in building industries. Concrete is a mixture of aggregates and paste [46]. The aggregates are gravel, sand, or crushed stone; the paste is water and Portland cement [47]. The use of concrete is developing with a superior performance concrete designed for specific construction and building requirements. Cement, a concrete ingredient, comprises 10 to 15% of the concrete mix by volume. Through a process known as hydration, the water and cement will harden and bind the aggregates to form a rocklike mass. Cement reacts with water in hydration and hydrolysis actions [48]. A dormant period occurs after the preliminary mixing and the first reaction climax, and prior to the concrete hardening. In structural engineering and civil engineering, serviceability is the condition under which a building will be useful. If the limit states are exceeded, then the structure will be weak. This relates to conditions different from the building strength that make the buildings unfit. Serviceability limit states in the design of structures include factors such as excessive vibration, cracking, deflection, fire resistance, overall stability, and durability. For satisfying the serviceability limit states, a concrete structure should be serviceable and function as expected during its service life. Extreme deflection must not impair the purpose of the structure or be aesthetically improper.

**Figure 1:**
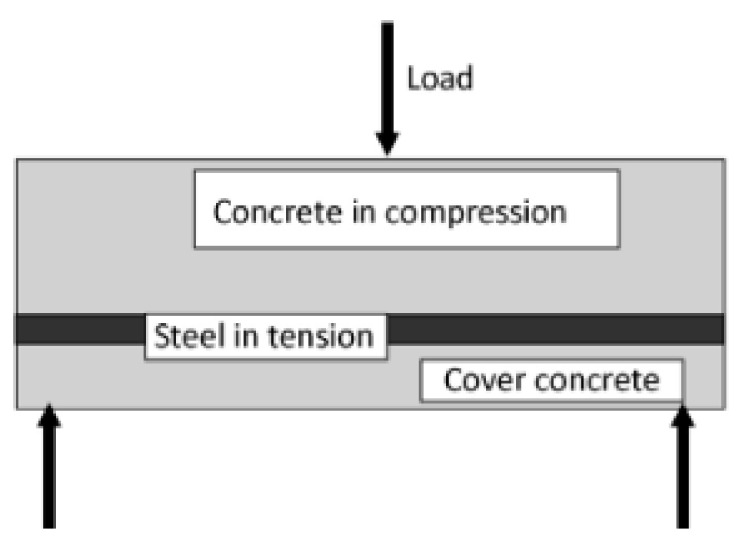
Schematic illustration of a simple reinforced concrete beam. Reproduced from [52].

**Figure 2:**
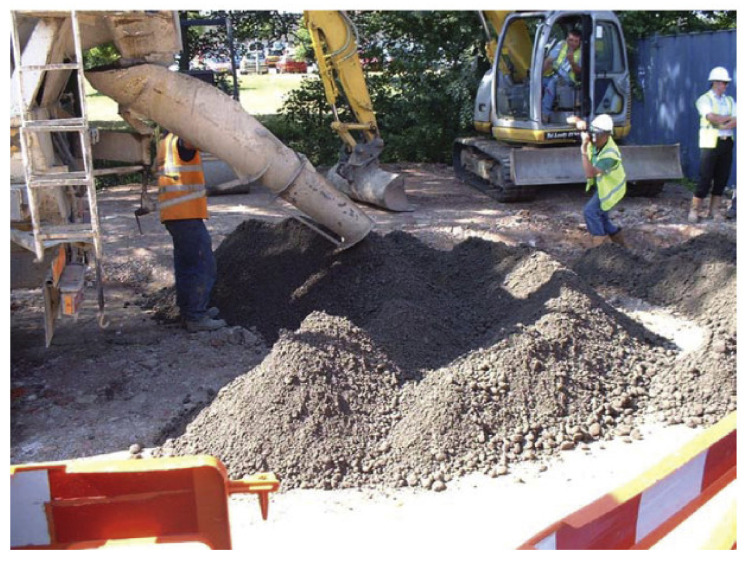
Semidry concrete for a road foundation. Reproduced from [52].

Cement manufacturers mine materials such as iron ore, shale, limestone, and clay; crush and screen the rock; and place it inside a cement kiln. After heating at higher temperatures, these materials will form a tiny ball termed as a “clinker”, which is extremely fine-ground to prepare Portland cement. Silica and lime make up about 85% of the cement ingredients. Other elements present are iron oxide and alumina [49]. Plasticizers are chemical compounds that help in concrete production with an almost 15% lower content of water. Superplasticizers permit a water content reduction of 30% or higher. Long-term creep prediction has been a main issue in designing concrete structures for some time [50]. Concrete creep is the structure deformation under sustained load. Long-term shrinkage and concrete creep can influence a concrete structure’s lifespan [51]. Reinforced concretes are composite materials made up of various constituent materials with distinct properties that complement each other [52]. For reinforced concretes, the component materials are mostly concrete and steel (Figure 1). Figure 2 shows semidry concrete that contains only enough water for the cementitious materials to hydrate and should be compacted using a roller. Current concrete structures require structural components that have improved mechanical properties and higher durability. This can be achieved by the incorporation of nanostructured materials in concrete materials that can improve their mechanical properties. The use of nanomaterials in concrete structures has been confirmed to provide increased durability and higher mechanical strength, which reduce their maintenance needs or any requirement for quick replacement.

Despite such progress in nanotechnology, proper information regarding nanomaterials’ environmental and human health impacts has been insufficient until now [53,54,55,56]. Thus, a careful approach must be developed when working with these materials. Nanosafety is considered as a growing concern, as the continuous exposure to the engineered nanomaterials is associated with many health effects, including pulmonary inflammation [57,58,59,60,61], genotoxicity [60], carcinogenicity [62], and circulatory effects [60]. As nanomaterials are difficult to detect as soon as they are released into the environment, they can lead to many types of environmental and health concerns if the remediation system is risky. Therefore, further studies are very important for systematically labeling the structure–function relationship of nanomaterials with regard to their basic chemistry. Moreover, complete risk evaluations must be performed on nanomaterials, which represent a real exposure hazard all during their manufacture or use [63,64,65,66,67,68]. Hence, green nanotechnology is also considered to lower the potential ecological and human health dangers from the manufacturing and use of nanosized materials, and to progress toward the replacement of predominant materials with innovative nanosized materials, which are extremely environmentally friendly [69,70].

**Figure 3:**
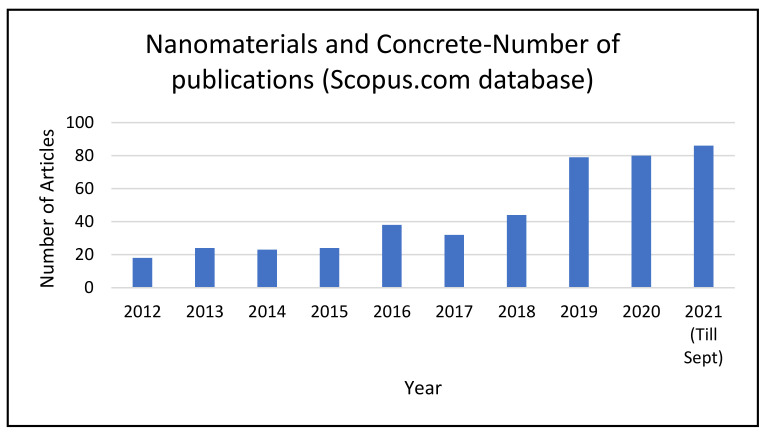
The number of articles (by year) in which ‘nanomaterials’ and ‘concrete’ were seen in the abstract, title, or keywords in the last 10 years. Data were obtained from the scopus.com database (20 September 2021).

**Figure 4:**
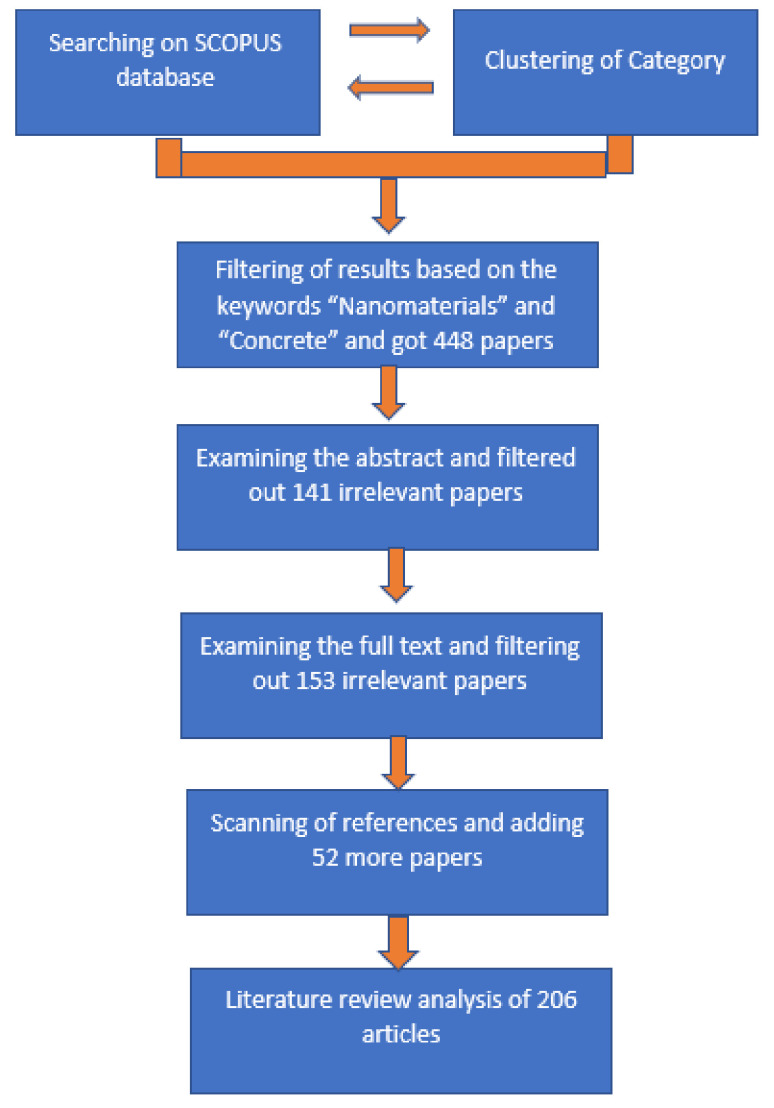
A flow chart demonstrating the steps of the literature review’s methodology.

**Figure 5:**
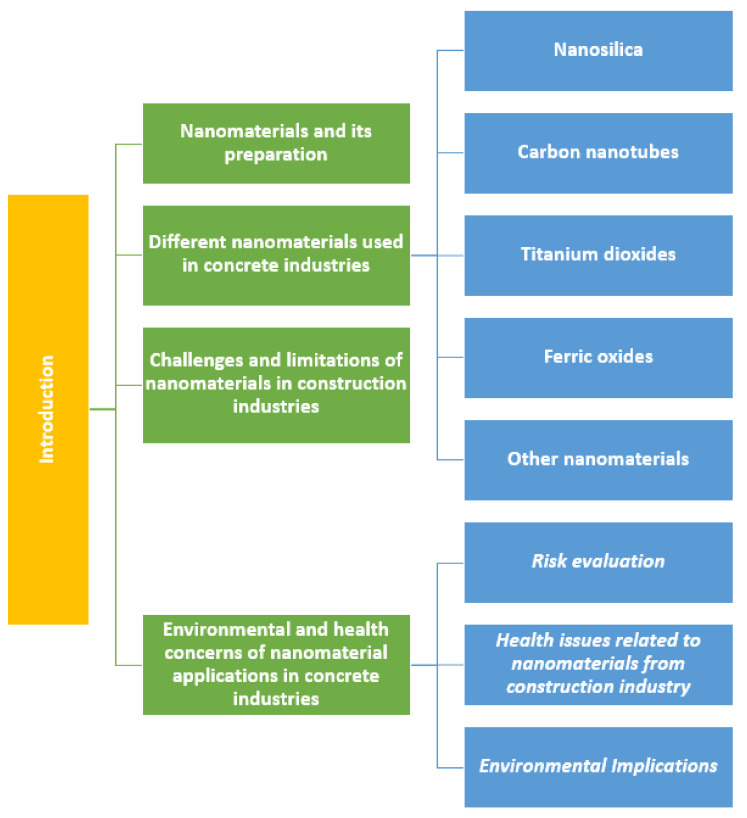
A schematic illustration of the study’s structure.

In the present study, we analyzed the use of nanomaterials in the prominent concrete sector field in the construction industries. The evolution of the number of publications related to the nanomaterials in concrete sector is presented in Figure 3, which was generated from the scopus.com database, and presents the number of studies carried out in each year over the period of last 10 years in which the expressions ‘nanomaterials’ and ‘concrete’ were found in the title, abstract, or keywords. The database returned 448 hits for the search with the keyword expressions ‘nanomaterials’ and ‘concrete’. In 2021, 86 publications have been reported so far, while in 2012, only 18 studies were reported. Thus, a clear growth in interest in nanomaterial application in the concrete sector was observed in the past 10 years. Out of the 448 papers found in the database, 252 were research studies, 82 were conference papers, and 17 were review papers. We have included 206 papers in this study based on their relevance, and removed the irrelevant papers. Figure 4 shows a flow chart demonstrating the steps of the literature review’s methodology. We analyzed different nanomaterials such as nanosilica, carbon nanotubes, titanium dioxide, ferric oxides, etc. used in the concrete application. Out of the papers obtained from the database, we selected the papers based on the materials and topics discussed in this study; for example, concretes based on nanosilica, carbon nanotubes, titanium dioxide, and ferric oxides; as well as the related challenges, limitations, and environmental and health concerns. Figure 5 shows a schematic illustration of the study’s structure.

An important challenge associated with the concrete sector is the decrease in the emissions of carbon dioxide gas. A solution to the aforementioned problem is the development of concrete frameworks with improved mechanical properties and enhanced durability, which would in turn reduce the maintenance needs and also reduce the requirement for early replacements. However, we also must consider the risk assessment, health issues due to the exposure of nanomaterials, and environmental implications associated with the application of nanomaterial-based concrete structures. The above mentioned positive and negative factors were the main motivation of the current study. We attempted to analyze whether the inclusion of nanomaterials in concrete structures would improve the mechanical properties (tensile strength, compressive strength, flexural strength), ductility, freeze–thaw resistance, and abrasion resistance of the concrete. We selected the nanomaterials (nanosilica, carbon nanotubes, titanium dioxide, ferric oxides, etc.) presented here based on the articles we obtained from the Scopus database for concrete application. Moreover, in the current study, we sought to analyze whether the inclusion of carbon nanotubes and titanium dioxide in concrete could offer self-sensing and self-cleaning properties to the concrete structures. Furthermore, we also analyzed the challenges and limitations of the application of nanomaterials in the concrete sector. The amount of manufactured nanomaterials continues to grow at an exponential speed, and conventional toxicity approaches cannot keep up with the total number of nanobased materials employed in the construction sector. Thus, in our study, the risk evaluation, health issues due to nanomaterial exposure, and environmental implications related to the application of the construction field nanomaterials were comprehensively examined. The important features to be examined and discussed include a toxicological analysis, long-/short-term exposure dangers of nanomaterials to the health of humans and environment in the service life of the material, and the dangers related to nanomaterial disposal.

To the best of our knowledge, studies on the up-to-date progress in nanotechnology for the application in concrete are still very limited. There is no study reported so far that collectively discussed the application of nanomaterials in the concrete sector, together with the challenges and limitations, the concrete recycling trend, risk assessment, health issues related to nanomaterials in concrete, and related environmental implications. This kind of study might be very beneficial to the appropriate development of applications and research interest toward additional progress in nanomaterials. This study aimed to contribute a comprehensive view of nanomaterials employed in the concrete sector and their possible toxicological implications. We understand that this study can contribute new knowledge as well as insights for the technical community in the concrete sector. The new and improved concrete structures with improved mechanical properties and enhanced durability can in turn reduce the maintenance needs and also reduce the requirement for early replacements. This research paper is expected to bring proper attention to the probable effects of construction waste, together with the importance of proper regulations, on the final disposal of the construction waste.

## 2. Nanomaterials and Their Preparation

Nanomaterials can be categorized according to their dimensions and size [10]. There are four categories of nanomaterials: zero-dimensional (0D), one-dimensional (1D), two-dimensional (2D), and three-dimensional (3D). Some nanomaterials occur naturally (e.g., sand); some are unintentionally developed (e.g., car exhaust fumes); and some other nanomaterials are purposefully made. However, particular care should be taken in the management of these materials. Naturally occurring nanoparticles are developed using a range of mechanisms, and can show a range of effects. Primary producers of nanomaterials include desert surfaces, volcanic eruptions, and cosmic bodies. Deserts can produce nanomaterials en masse, with approximately 50% of all aerosols in the troposphere developing from deserts, comprising a variety of materials signified in mass distributions. Toxic materials such as polycyclic aromatic hydrocarbons, mercury, and cadmium were found in an analysis conducted in China and South Korea in the course of a dust storm [43].

Engineered nanoparticles are developed through either a bottom-up approach or a top-down approach. The two approaches vary such that in the top-down preparation approach, larger structures will be gradually reduced in size to nanometer dimensions, whereas the bottom-up preparation approach involves developing nanoparticles with individual atoms or molecules until the required size is accomplished. Additional categorization can be made depending on the mechanism of the preparation process, classifying most engineered nanomaterials into three categories: physical, mechanical, and chemical processes. Nanosilica is a nanomaterial commonly used in the concrete application, and has characteristics such as smaller particle sizes, good dispersion, high chemical purity, large surface energy, stronger surface adsorption, and large specific surface area [71]. Various methods, such as pressurized carbonation [72], combustion in a diffusion flame [73], chemical vapor deposition [74], plasma synthesis, chemical precipitation method, and the sol-gel process are used for preparing silica nanoparticles. Out of the above-mentioned fabrication methods, the sol-gel process is a superior method that can be followed for preparing superior-quality, narrow-size-distributed, spherical nanoparticles. In addition, nano-SiO2 can be prepared by grinding rice husk ash finely to develop nanomaterials [75]. Carbon nanotubes (CNTs) are also commonly used for concrete application, and the tensile strength of CNT is almost 100 times higher than that of steel with a similar diameter. CNTs also have a higher thermal conductivity, similar to that of diamond crystal [76]. High-temperature manufacturing methods such as laser ablation or arc discharge were initially employed for producing CNTs [77]. However, these techniques have been replaced by the lower-temperature chemical vapor deposition method. This is because the density, purity, diameter, length, alignment, and orientation of the nanotubes can be precisely controlled with this method.

Recently, titanium dioxide (TiO_2_) nanoparticles have been used for concrete application, and they have been prepared using different methods such as electrochemical synthesis [78], microemulsion-mediated methods, wet-chemical synthesis by precipitation of hydroxides from salts, gas phase (aerosol) synthesis [79], metal organic chemical vapor deposition (MOCVD) [80], the sol-gel process, and reverse micelles. The larger effective surface of TiO_2_ nanoparticles helps to absorb a large quantity of water, leading to a reduction in the concrete’s workability [81]. Polycarboxylates are one of the nanomaterials employed in concrete [82]. Generally, polycarboxylates comprise a methoxy-polyethylene glycol copolymer reinforced with methacrylic acid. The carboxylate groups contain a water particle, contributing a negative charge along the polycarboxylates’ backbone. The polyethyleneoxide group provides a nonuniform distribution of the electron cloud that can provide a chemical polarity to the secondary reaction. Typically, polycarboxylates will be applied in concrete as a high-range water reducer, and this incorporation helps to control the workability of the concrete at a lower water-to cement-ratio [83].

Incidental nanomaterial generation takes place by different channels that are not directly noticeable in everyday life; however, they can be obvious when recognized, as well as addressed. A perfect example of incidental nanoparticles that develop in the built environment are those produced by cars through exhaust emissions, brake pad material loss, tire material loss, and paint deterioration. Presently, one of the major challenges related to nanomaterials is inhalation exposure, in spite of its numerous remediation application [84]. Moreover, the nanomaterial preparation process can be difficult and complicated. In addition, the complete process is expensive, a requires optimum results—particularly concerning their use in different consumer goods—to avoid financial losses. Nanomaterials that are engineered might also end up in water bodies, prior to their accumulation into large-sized particles. Even though an engineered nanomaterial concentration is expected to be very small, frequent discharge might lead to an increase in concentrations over time, worsening the related adverse impacts.

The nanomaterial field is highly interested in increasing the sustainability of the processes involved in nanomaterial production, which stimulates the assessment of alternate inputs for nanoparticle production and the application of green synthesis methods. Green nanotechnology regards the manufacturing processes that are environmentally sustainable and economic. Using clean manufacturing processes for yielding value-added materials from waste materials has arisen as an appropriate synergy to achieve both circularity and sustainability goals. For reducing the adverse ecological impacts and health risks related to the manufacture, use, and discarding of novel nanomaterials, the materials engineering and nanotechnology fields are highly concerned with sustainability methods, metrics, and frameworks. Currently, green nanotechnology is expected to progressively impact on a large range of economic sectors.

## 3. Nanomaterial Application in Concrete Industries

**Figure 6:**
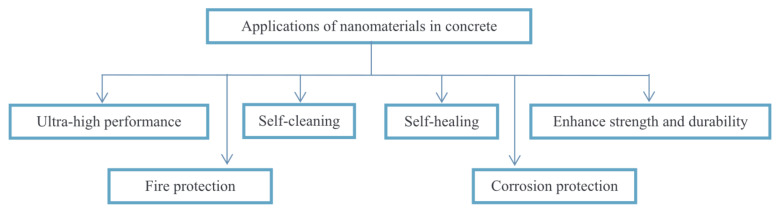
Applications of nanomaterials in concrete. Reproduced from [33].

Concrete has been extensively reviewed in several research works as a nanoenabled material, with many studies confirming the utilization of several nanomaterials. Figure 6 shows the applications of nanomaterials in concrete [33]. There is a relation between the bulk properties and microstructure, and therefore researchers are continuously trying to establish this relationship in concrete. In the following section, we will discuss the application of different nanomaterials such as nanosilica, carbon nanotubes (CNTs), ferric oxide, and nano-titanium oxide in concrete industries. It can be noted that majority of nano-based concrete products are still at the laboratory-scale level, and only a few products, such as nanosilica particles (Gaia) and polycarboxylate nanoproducts, have been commercialized and are used by the industry. Nanoparticle-incorporated concrete is also known as nanoconcrete, and more commercialization is required.

### 3.1. Nanosilica

Nanosilica is nanomaterial used for construction applications that can substitute silica fume and microsilica fume. In fact, the more broadly used nanomaterial in concrete is silica. In typical concrete, silica exists within the framework of a standardized mix. The nanosilica reacts with lime at the time of the cement hydrating procedure as well as subsequently generating a C–S–H gel, which might enhance the mechanical properties and durability of the concrete. In some of the research works, it was observed that nanosilica utilization in concrete enhanced the particle packing [85]. The cement hydration rate also was improved, which efficiently improved the strength, and lowered the dormant period and time of setting. This nanomaterial also decreased the concrete porosity and prevented the possibility of the degradation of concrete [86].

Nanomaterials decrease the porosity of cement, producing a denser interfacial transition zone [87]. Concrete technology presents a higher extent of porosity, which is the fraction of material volume occupied by pores. A higher-strength concrete grade shows an increased density, less porosity, and very closed pores in, and lowers the carbon dioxide transmission coefficient in the concrete. Therefore, the carbon dioxide quantity transmitted into the concrete is consequently reduced. A higher strength grade of concrete requires higher consumption of cement and more hydration products in the hydration reactions, which assist the system in absorbing more carbon dioxide. Singh et al. [88] stated that the technique employed for incorporating nanosilica into cement composites can impact the porosity and mechanical properties of the composites. Nanosilica-included cement pastes were examined to understand the hydration process, as well as the microstructure evolution. Cement mortar with properly dispersed nanomaterials will have a denser microstructure even though the nanomaterials are incorporated in smaller quantities; however, if the nanomaterials are not well dispersed, this will lead to the formation of voids and weak zones. A bad dispersion of nanosilica in concrete materials can produce voids and weak zones, changing the material’s mechanical characteristics.

Li et al. [89] analyzed the properties and microstructure of superior-performance concrete, which is formulated by using particles of nanosilica and nanolimestone. The team employed Portland cement type I, as well as fly ash, with the binding agent’s silica fumes. The percent of nanolimestone and nanosilica by cement weight were noted to be 2.00%, 3.00%, and 4.00%, and 0.50%, 1.00%, 1.50%, and 2.00%, respectively. Considering the microstructure features, the results confirmed that the nanosilica acted as an efficient filling material that decreased the porous areas and enhanced the hydration process of the cement. It was noted that the tensile strength and compressive strength of concretes inclusive of nanosilica and nanolimestone were increased with respect to concretes with no additions. The results from this study confirmed that the nanosilica and nanolimestone contents were very important to the performance of the ultrahigh-performance concrete matrix.

In a study by Najigivi et al. [90], the team assessed the effects of two different nanosilica particles with different ratios on the compressive strength and workability, and of prepared binary blended concretes cured in lime solution and water as two distinct curing media. Test results for hardened concrete showed that the optimum replacement level of cement by N series of nanosilica particles for developing concrete with remarkably increased strength was set at 1.0 wt %, subsequent to curing in water. This study confirmed that nanosilica particles performed an important role in the concrete’s mechanical properties through the development of calcium silicate hydrate gel at the time of treatment. This played a significant role in increasing the binary blend’s compressive strength. A study by Barbhuiya et al. [91] reviewed the nanosilica preparation for concrete application, and the influence of the addition of nanosilica to cement concrete with regard to various components, hardened state, and fresh state; as well as physical properties such as setting times and workability.

Thus, overall it was noted that the nanosilica reacted with lime at the time of the cement hydration process, and subsequently enhanced the mechanical strength and durability of the concrete. In addition, we found that nanosilica and nanolimestone contents were very important to the performance of an ultrahigh-performance concrete matrix, due to the fact that their inclusion could improve the tensile strength and compressive strength of concrete. Moreover, the freeze–thaw resistance and abrasion resistance of the concrete could also be improved with the inclusion of nanosilica in the concrete mix. When increasing the nanosilica concentration, the flexural strength, split tensile strength, and compressive strength of concrete increases because the nanosilica functions as an activator to promote the hydration, thereby improving the microstructure pores. Furthermore, nanosilica can improve the interfacial transition zone, thereby changing the concrete matrix to be more dense, and thereby improving the concrete’s durability. Although several studies have been performed that confirmed the application of nanosilica as a suitable material in the construction sector, this nanomaterial has still not achieved momentum in the production of concrete. This is due to the formation of agglomerates, the unavailability in several nations, and the expensive nature. Another matter of concern is the absence of appropriate dispersion of nanosilica in concrete. Although the sonication method is a potential approach to better dispersion, alternate techniques should also be considered. This nanomaterial application is still in its investigation stage, and is developing from fundamental investigation to commercial applications. However, Gaia is one of the first commercial nanoadmixtures employed in concrete, and it was prepared by Ulmen S.A. (Santiago de Chile, Chile) and Cognoscible Technologies (Chile) for substituting silica fume in ready-mixed concrete amenities certified by the ISO 14001-Environmental Management System [92]. This product is in liquid form, which can efficiently disperse the nanosilica particles present in concrete. Gaia shows the perfect impacts of slump improvment, as well as water lessening, on the concrete. The self-compacting concrete design turns out to be a simple task with the benefit of the Gaia. The Gaia application at 1.3 wt % concentration contributed a slump loss of 30% in 1.5 h at a 20 °C ambient temperature for concrete. In the meantime, incorporation of Gaia contributed approximately a twofold rise in the compressive strength of concrete at 7 days and 28 days and was almost three times greater than the earlier compressive strength (at 1 day of age) of the reference concrete.

### 3.2. Carbon Nanotubes (CNTs)

Several studies have confirmed that carbon nanotubes in a lab environment can be used to improve the strength of materials and to contribute electrical conductivity [93]. Nanomaterials with properties such as strength and durability are of special interest in the manufacturing of concrete [43]. CNTs can be incorporated into concrete; however, their commercial utilization is not very advanced because CNTs are costly when considering the amounts needed for their inclusion in mass concrete, even though characteristic suggested proportions are just 1 wt %.

Presently, carbon nanotubes are also added to concrete as a nanofiller because they possess a higher surface area and unusual mechanical characteristics. Mohsen et al. [94] examined the influence of carbon nanotube utilization on the microstructure, permeability, strain capacity, and flexural strength of concrete. The test results confirmed this nanomaterial increased the concrete beam ductility by approximately 150%. A qualitative microstructural analysis showed the CNT filaments’ uniform dispersion inside the concrete hydration products in entire batches. The study confirmed that a large void content was occurring due to the increased surfactant content present in the solution after the sonication process. Gürkan et al. [95] demonstrated that relative to carbon nanotubes, carbon fiber utilization remarkably enhanced the load-carrying capability and ductility, leading to bending mode of failure even with inadequate shear reinforcement. In a study by Adhikary et al. [96], the impact of CNTs and graphene nanoplates on lightweight concrete properties were investigated. The test results confirmed that the compressive strength of lightweight concrete specimens was marginally increased by including CNTs and graphene nanoplates. The composite specimens fabricated with CNTs showed increased compressive strength relative to the concrete specimen fabricated with graphene nanoplates at the same concentration.

Lushnikova et al. [97] analyzed the impact of different carbon nanotubes when included in cement samples. CNTs affected the nanoscale processes in the C–S–H structure on the molecular level that kept the cementitious matrix together. The team employed molecular dynamics simulations for determining the impact of carbon nanotubes on the mechanical properties of C–S–H, such as elastic constants, bulk modulus, shear modulus, and the Poisson ratio. The findings of the simulations confirmed an increase in the examined mechanical characteristics. Consequently, this study confirmed that carbon nanotubes were nanomaterials that could improve the concrete’s mechanical properties. A study by Chandran et al. [98] proposed that the macroscopic carbon nanotube bars could be used as reinforcement bars for concrete structural elements, and this offered superior performance concrete that was crack-free and had increased ductility. In a research work by Yu et al. [99], the group examined the mechanical improvement in carbon-nanotube-reinforced concrete. The authors studied the interplay between calcium silicate hydrate and CNTs. This study confirmed that the porous property of C–S–H nanobranches could allow the CNT incorporation.

**Figure 7:**
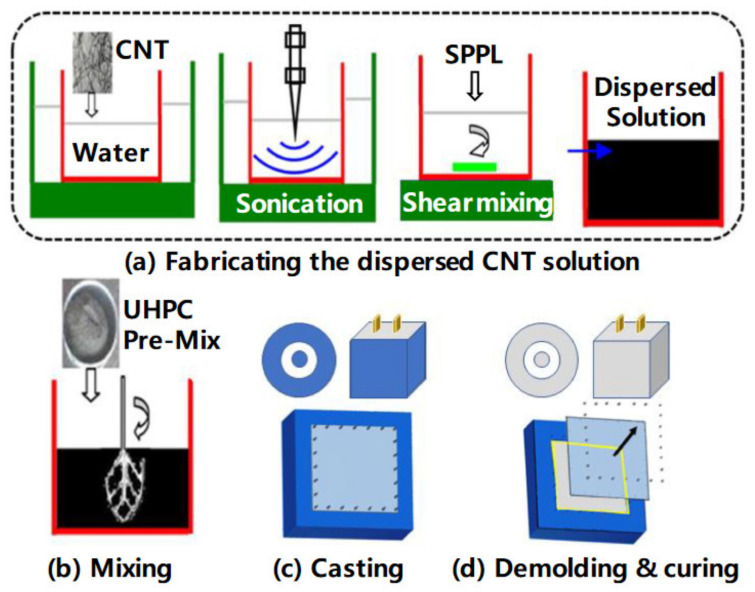
The specimen preparation process for CNT-incorporated ultrahigh-performance concrete. Reproduced from [100].

**Figure 8:**
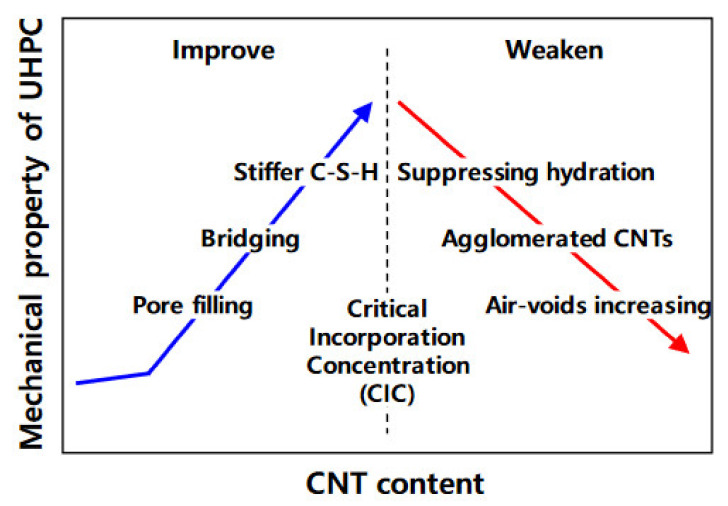
The effects of CNTs on the mechanical properties of ultrahigh-performance concrete (UHPC). Reproduced from Ref [100].

Ultrahigh-performance concrete shows increased crack resistance and durability, with 150 MPa compressive strength, and thus it could be employed for the construction of facilities requiring adequate physical protection. CNT incorporation in ultrahigh-performance concrete was examined by Jung et al. [100] with respect to mechanical properties and electromagnetic shielding effectiveness. Figure 7 shows the preparation process of the specimen, consisting of four processes: (1) preparation of dispersed CNT solution; (2) mixing of the ultrahigh-performance concrete dry premixture with the solution; (3) casting; and (4) curing. Figure 8 shows that dispersing CNTs in ultrahigh-performance concrete could improve the mechanical properties up to the critical incorporation concentration due to the bridging effect, pore filling effect, and development of a hydration product with increased stiffness. The results confirmed that the CNTs considerably improved the electrical conductivity, and led to shielding effectiveness up to the percolation threshold. Hawreen et al. [101] examined the impacts on the long-term creep, as well as concrete shrinkage, due to the incorporation of various types of carbon nanotubes. The team employed concrete with 0.05% to 0.5% of functionalized and unfunctionalized carbon nanotubes and 0.35–0.55 water-to-cement ratios. With CNT incorporation, the concrete’s compressive strength improved to 21%. Incorporating carbon nanotubes resulted in a decrease in the early and long-term shrinkage of the concrete of 54.0% and 15.0%, respectively. Here, the carbon nanotubes basically influenced the stiffness and porous structure of the cement paste, which covered about 30% of the concrete volume. The CNT addition to concrete showed 17.0% to 18.0% lower long-term creep as compared to the concrete with no CNTs. CNTs have been considered as advanced nanomaterials in the civil engineering sector that can reduce nanocrack development. The presence of CNTs in concrete might improve the flexural strength and compressive strength of the concrete.

Thus, it was noted that the carbon nanotubes were nanomaterials that could improve the concrete’s mechanical properties such as compressive strength, flexural strength, and ductility, and also offered crack resistance and increased electrical conductivity. The carbon nanotube application can increase the concrete beam ductility by approximately 150%. Moreover, the compressive strength of lightweight concrete specimens can also be increased by including CNTs. For improving the different properties of cement-based composites by the addition of CNTs, the appropriate dispersion of CNTs must be confirmed for the manufacture of CNT-containing cement-based composites. The knowledge of the chemical–mechanical dispersion mechanism of CNTs in cement-based composites is definitely beneficial in the design of CNT-containing functional cement-based composites. Various dispersion techniques, such as ultrasonication, use of admixtures or siliceous materials, minimization of the water-to-cement ratio, and modification of CNTs, have been attempted to disperse CNT particles in cementitious composites. The admixed carbon nanotubes could also reduce the porosity of the cementitious matrix. Furthermore, CNT incorporation into concrete can result in a decrease in both the early and long-term shrinkage of concrete.

### 3.3. Titanium Dioxide (TiO_2_)

Another nanomaterial used in concrete is titanium dioxide. The inclusion of titanium dioxide in concrete can offer concrete some self-cleaning ability. Titanium dioxide-incorporated concrete can permit a photocatalytic degradation of contaminants from automobile and industrial emissions. TiO_2_ is manufactured in great quantities due to its photocatalytic, stable, and anticorrosive properties. The photocatalytic ability of titanium dioxide results from the higher surface area of the nanoparticles; thus, when incorporated in concrete, the material turns out to be self-disinfecting and self-cleaning. In the presence of light, the aforementioned nanomaterial disintegrates the dirt and organic pollutants present on the concrete surface into carbon dioxide and water; the catalytic reaction products are then simply detached by rain or simple cleaning. The addition of nano-TiO_2_ in cement matrices can offer concrete frameworks with self-sensing and self-cleaning capabilities. Zanfir et al. [102] studied the sol-gel preparation, as well as characterization of a titania–silica fume composite generated by the coating of silica fume particles with TiO_2_ photocatalytic nanoparticles. It was noted that the TiO_2_–silica fume composite powders demonstrated an improved pozzolanic activity and could be employed for the partial replacement of Portland cement in the self-cleaning mortar preparation. In a research work by Nikbin et al. [103], the group examined the shielding performance of heavy concrete with magnetite aggregates and different concentrations of TiO_2_ nanoparticles, substituted by weight of cement in different temperatures cycles. The test results confirmed that samples with 6% TiO_2_ nanoparticles met the design requirements (increased compressive strength).

**Figure 9:**
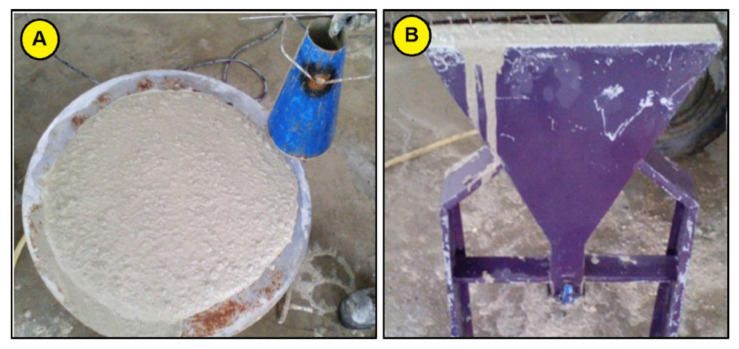
Experiments on fresh concrete: (**A**) slump flow test apparatus; and (**B**) V-funnel test apparatus. Reproduced from [81].

The Jubilee Church in Rome, Italy, which was constructed in 2003, was one of the first buildings that employed reinforced and self-cleaning concrete [104]. The construction possessed three iconic shells built in 2001–2002 that used precast panels with photocatalytic titanium oxide nanoparticles. The titanium oxide nanoparticles can absorb energy from light and use this energy for accomplishing the pollutant photocatalytic degradation. Joshaghani et al. [81] examined and compared the impacts of various nanoparticles, such as TiO_2_ nanoparticles, alumina nanoparticles, and nano-ferric oxide, on a self-consolidating concrete’s performance regarding durability and mechanical properties by performing various experiments. Self-consolidating concrete is a concrete type that can be placed, as well as compacted under its own weight, with no requirement for compaction. Figure 9 shows the method of performing the V-funnel and slump flow tests. The incorporation of nanomaterials in the mixes moderately reduced the V-funnel time, confirming that the flowability property of the self-consolidating concrete increased marginally. This study confirmed that partial substitution of cement with nanomaterials on average increased the durability and compressive strength of self-consolidating concrete, but also resulted in a workability reduction.

From the analysis of different studies, it was observed that the incorporation of nano-TiO_2_ in cement matrices could offer concrete frameworks with self-sensing as well as self-cleaning capabilities. It could also contribute improved mechanical properties, such as the compressive strength and durability, to the concrete. Moreover, incorporation of nano-TiO_2_ to the concrete mix could moderately reduce the V-funnel time, thereby confirming the improvement in the flowability property of self-consolidating concrete. The TiO_2_ nanoparticles also improved the strength and the durability of the nano-based concretes by enhancing the carbonization tolerance, as well as the resistance to different chemical attacks, which was additionally due to the upgrading in the microstructures, porosity reduction, and refinement of the voids or microcracks. The larger surface area and stronger reactivity of the nanoparticles added into the concrete structures could improve the pozzlanic reactions, leading to a significant improvement in strength performance.

### 3.4. Ferric Oxide (Fe_2_O_3_)

**Figure 10:**
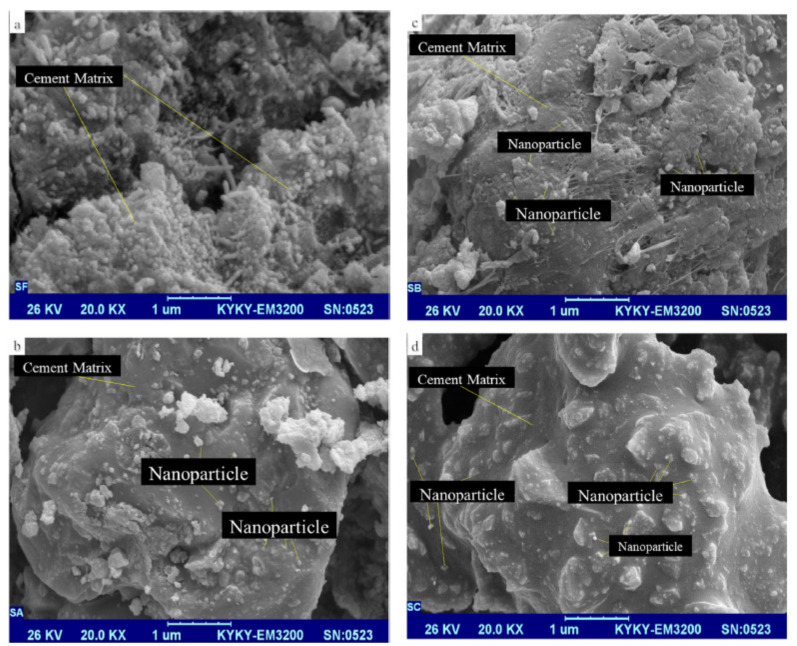
Scanning electron microscopy images of cement composites with: (**a**) 0.0 wt %, (**b**) 2.0 wt %, (**c**) 4.0 wt %, and (**d**) 6.0 wt % of ferric oxide nanoparticles. Reproduced from [105].

The optimum incorporation of ferric oxide nanoparticles in concrete specimens might increase the compressive strength. Kani et al. [105] studied the properties of cement composites and mortars in the presence of ferric oxide nanoparticles as the modifier. The cement composite was synthesized by the sol-gel technique with the complex ligand tetraethylammonium orthosilicate. Ferric oxide nanoparticles were incorporated at 2.0, 4.0, and 6.0 wt % concentrations in the cement composite; whereas for mortars, 2.0, 3.0, and 4.0 wt % of ferric oxide nanoparticle concentrations were employed. Figure 10 presents the scanning electron microscopy images of the cement composite with (a) 0.0 wt %, (b) 2.0 wt %, (c) 4.0 wt %, (d) and 6.0 wt % of ferric oxide nanoparticles. The test results confirmed a variation in the molecular structure and phase composition of the cement composites, resulting in stronger bonds in the silicate network, with an extremely ordered arrangement of nanomaterials in them. The mechanical properties of mortar samples increased with inclusion of ferric oxide nanoparticles, and a 3.0 wt % dosage was recognized as the optimal concentration.

A study by Heikal et al. [106] examined the influence of ferric oxide nanoparticles on the properties and durability resistance to chloride and sulfate anion attacks. Ferric oxide nanoparticles were manufactured from the heating of Fe(CH_3_COO)_2_OH at 450 °C and 300 °C at a 2 h soaking time. The manufactured ferric oxide nanoparticles demonstrated the buildup of fine ferrihydrite and hematite crystals with an almost 10–20 nm grain size. Ferric oxide nanoparticles increased the durability of composite pastes containing ferric oxide nanoparticles toward chloride and sulfate anion attacks. Mixes with 0.5–1.0% ferric oxide nanoparticles confirmed low values of total chloride and total sulfate contents. This was due to a finer and denser matrix with low porosity, which blocked the empty pores to the diffusion of chloride and sulfate anions, and therefore demonstrated increased values of bulk density and compressive strength.

Thus, overall, it was noted that the addition of ferric oxide nanoparticles to the concrete could improve its mechanical properties, such as its compressive strength. Further, the hardened concrete’s water absorption could also be improved with the inclusion of iron powder. Moreover, it was observed that the ferric oxide nanoparticles could increase the durability resistance of concrete against chloride, as well as sulfate anion attacks.

### 3.5. Other Nanomaterials

Polycarboxylate, a nanomaterial, is an admixture in concrete. The construction of remarkable concrete structures have been benefited from the exceptional performance of present commercial polycarboxylate products, including the construction of the tallest skyscraper in the world—Burj Khalifa, Dubai, with an 820 m height. The development of higher-strength concrete, including a kind of polycarboxylate product and a retarder, permitted the structural concrete to be pumped up to an increased height of 650 m, and showed slump retention over 3 h at 50 °C for the concrete [107]. The stress-free handling guideline or technique has permitted polycarboxylate to become a popular nanomaterial used in cement-based materials.

Moreover, an increasing interest in the use of cellulose nanomaterials as fillers and reinforcement in concrete has been noted [108]. Test results confirmed that an increased elastic modulus was obtained, retaining a similar water-to-powder ratio. Types of cellulose commonly used in concrete are cellulose filaments, microfibrillated cellulose, cellulose nanocrystals, and cellulose nanofibrils. For nanocellulose, promising results have been presented, and in a study by Barnat-Hunek el al. [108], the team analyzed nanocellulose from carrot and apple in the form of nanofibers and nanocrystals. It was noted that increased concentration of nanocellulose in the concrete resulted in a higher tensile strength in bending.

Table 2 presents the different functions of nanomaterials when added to concrete [109,110,111,112,113,114,115,116,117]. It is evident that it could improve the mechanical properties and overcome physical and chemical deteriorations.

As we can note, although nanomaterials offer superior properties to concrete, the majority are not used as much commercially, due to their limited availability, along with the increased price of the nanomaterials. However, global commercial interest in CNTs is reflected in their manufacturing capacity, which currently is more than several thousand tons/year [118]. In 2015, Zeon Corp. (Tokyo, Japan; www.zeon.co.jp, accessed on 5 October 2021) opened the world’s foremost mass-production facility for high-grade CNTs at its Tokuyama facility in Shunan City, Japan. This facility produces CNTs using the Super Growth (SG) method, and they have greater than 99% purity [119]. Table 3 presents an overview of the improvements in concrete properties with the addition of nanomaterials.

So far, there have been no nanomaterials employed commercially in the construction sector, except for nano-SiO_2_ and polycarboxylate. However, these nanomaterials are not very economical or as abundantly available as other supplementary cementitious material. However, if thorough research is carried out in this area, then there will be an opportunity to develop advanced, highly performing, durable, economical, and sustainable concrete. Nano-SiO_2_, carbon nanotubes, and titanium dioxide have good possibilities for use in nanoconcrete. Smart concrete and self-sensing concrete tend to find a suitable space in the construction sector. The application of specific nanostructured materials could improve the performance and the life cycle of concrete structures. Scientists are attempting to develop the manufacturing technology for producing nanomaterials at a commercial scale so that these materials will be easily accessible economically.

## 4. Challenges and Limitations of Nanomaterials in Construction Industries

Specific consideration must be given to the probability of penetration of water within the building matrices with the utilization of nanomaterials [120,121,122,123,124,125]. The clay-based nanomaterials are hydrophilic, and hence special care must be taken for controlling the water necessities in the clay–cement composite. A reduction in water can be accomplished through an organic cation exchange modification, in which the organic cation replaces calcium or sodium existing in the interlayer, lowering its hydrophilicity. In the recent past, the chemical binding of polyvinyl alcohol has been employed for creating linked clay particle chains that, while incorporated in the cement, could enhance their performance [126]. When nanocoatings were employed in some cultural heritage stone, the major problem to be considered was their photoinduced super hydrophilicity, which could adversely impact the stone [127].

Another major challenge in using nanoparticles in the construction industries is the absence of their homogenous dispersion [128,129]. The nanoparticles are inclined to agglomerate in a cement matrix, and hence could not be homogeneously dispersed with a basic mixing technique [130]. Mostly, carbon nanotubes/carbon nanofibers have increased hydrophobicity and stronger self-attraction. Including these nanomaterials in the construction industries appears extremely attractive, but somewhat complicated. For solving this problem associated with dispersion, proper knowledge of the intricate mechanisms in the paste and the interaction at interfaces are important parameters for optimizing the inclusion of carbon nanotubes/carbon nanofibers in concrete [126]. Extra steps might be needed when mixing these materials, such as purification and functionalization prior to mixing. These may still cause weak bonding between the cement and the nanomaterials (carbon nanotubes/carbon nanofibers). Some scientists have concentrated on overcoming this issue by introducing a simple technique for the development of better dispersion of carbon-based nanomaterials.

Nanomaterial compatibility with building materials is another limitation in the application of nanomaterials [131,132]. Specifically, regarding titanium dioxide nanoparticles, some research has confirmed that when titanium dioxide is added to cement, there might be additional complexities, as compared to when titanium dioxide is coated on other substrates such as ceramics and glass. Cement has poor stability and less surface area [133,134], which are adversative to the photocatalytic reaction, and lead to a negative effect regarding the use of poor stability in building matrixes. As time passes, its efficiency reduces. Mainly, after four months, the photocatalytic influence appears to drop irrespective of whether it was mixed with the bulk or used as a coating on the surface.

Another drawback of the use of nanoparticles in the construction sector is the fact that at the time when nanomaterials are employed for enhancing the strength, the materials with higher strength also have a higher density, which results in greater structure weight [135].

The high cost of nanomaterials is an extremely significant drawback in the use of nanomaterials in the construction sector [136,137]. This is due to the uniqueness of this technology and the intricacy of the equipment employed for manufacturing and characterization practices. However, expenses have been shown to be reduced with time, and as fabrication technologies progress, these expenses might be reduced further. Presently, the greater expense of the nanostructure-based self-healing concrete, as compared to the conventional ordinary Portland cement-based concretes, is mostly due to their superior qualities, restricted knowledge as regards their implementation, as well as less manufacturing globally. In spite of the high price of the nanomaterial-based self-healing concretes, the implementation of these materials must be considered on account of their long-term benefits. It is expected that nanomaterials will contribute exceptional solutions for solving any complex issues, resulting in commercial scale uses, and thus making them economical [138]. Excluding all these, certain fundamental disadvantages of the use of nanomaterials in the construction sector include environmental problems and health hazards [139,140,141,142,143]. Nano-enabled construction products can be dangerous to human health [144,145,146,147]. Constructive structure is presented in the natural surroundings, and hence all the materials employed in the facilities are required to be compatible with the natural surroundings with almost zero environmental impact, as feasible. The most frequent potential issues include the discharge of nanomaterials through dust into the air, nanomaterials invading the groundwater, and exposure to possibly dangerous materials at the time of construction and during maintenance procedures.

Numerous factors should be considered for developing nanomaterial-based concretes. First of all, the concrete and associated materials should be fabricated at a commercial scale. Although the expense of high-priced concrete structures turns out to be lower, it should have the capability of handling enormous materials in an ecofriendly and safer way. Second, advances need to be methodically established with field assessments to achieve the proper knowledge, as well as assurance in the concrete field. Finally, concrete structures are difficult to destroy and require greater energies or explosives to break up. Therefore, nanomaterial-based concrete fabrication must be compatible with the above-mentioned traditional practices.

## 5. Environmental and Health Concerns of Nanomaterial Applications in Construction Industries

**Figure 11:**
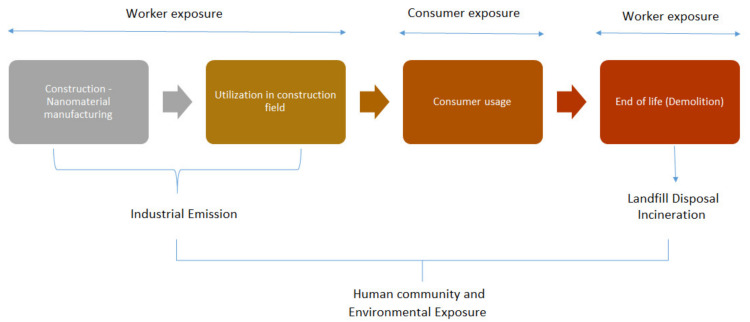
Potential exposure routes during the complete cycle of construction nanomaterials.

The advancement and progress of nanotechnology can upgrade the properties of building/construction materials, and thus assist sustainability [148,149,150,151]. There are obvious benefits related to the development of several nano-enabled products; however, there might also be dangers. For example, several health hazards are involved in the utilization of certain nanomaterials. Several studies have examined the negative effects of nanomaterials on human health and on the surroundings [152,153,154,155,156]. Figure 11 presents the potential exposure routes during the complete cycle of construction nanomaterials. The precise form, as well as the dose, of the nanomaterials involved are significant factors in whether the danger is substantial, making it important to gain a better knowledge of the materials being used. Analyzing the inadequate data accessible in the public domain has shown that it is difficult to be certain about the utilization of nano-enabled products in the construction field and their market penetration. Further, it is more difficult to predict which new advancements in nanomaterials might become commercially obtainable for products in the immediate future, and how extensively they may be used. The construction/building industry is continuously considering different techniques for improving economic and environmental sustainability. Although using nanomaterials in concrete has enhanced constructability and has resulted in significantly upgraded construction features, there still exists the problem of recycling these nanomaterials, together with the health dangers related to them to the laborers in construction/demolition and members of the societies in the area [157].

### 5.1. Risk Assessment

Risk assessment examines the hazards to the health of humans and the environment of a sole substance at a specific point in its production time, service life, or discharge [158,159,160]. Risk assessment is regularly carried out to recognize if any stages of life cycle present a risk. Recently, a momentous enhancement in the usage of nanomaterials in different construction materials was noted that was inclusive of coatings, glass, mortar, timber, bricks, steel, and concrete [161,162,163]. Partial scientific data has been collected that supports or discourages nanotechnology implementation in the construction industries. The important features to be analyzed and discussed include a toxicological analysis, longer-period and shorter-period exposure dangers of nanomaterials on the health of humans and environment during the service life of the material, and the dangers related to the nanomaterials’ disposal. There were several previous studies carried out for the risk evaluation of nanomaterials used in the construction industries [164,165,166,167,168,169].

### 5.2. Health Issues Related to Nanomaterials from Construction Industry

During the utilization of nanomaterials in the construction industry, exposure can lead to the inhalation of nanomaterials, or their absorption by straight contact with exposed skin. Consequences of nanomaterial exposure are not adequately realized, thus demanding studies to understand the interactions between nanomaterials and organic materials. Research works on the intake of many standard nanomaterials by mice generated understandings of the possible adversarial influences of nanomaterial exposure; however, generalization of the results was restricted by the experiment’s subjects [170,171,172]. During recent years, toxicological examinations have been carried out with various kinds of engineered nanomaterials to understand their impact on the human body. Some of the health effects of nanoparticles used in the construction industry on human organs are presented in Table 4 [173,174,175,176,177,178].

In relation to the most commonly used nanomaterials in the construction industry, it can be noted that their risks are different. Several nanomaterials are comprised in the venture that the Organization of Economic Cooperation and Development (OECD) has started to promote the complete characterization of nanomaterials already present in the market or soon-to-be commercialized [179]. Steffen et al. [179] presented an examination of the published OECD reports regarding the information on ecotoxicology, environmental fate, and the chemical and physical properties. The list includes gold nanoparticles, nanoclays, dendrimers, zinc oxide, cerium oxide, aluminium oxide, titanium dioxide, silver nanoparticles, multiwalled carbon nanotubes, single-walled carbon nanotubes, and fullerenes. In the USA, as per the National Institute for Occupational Safety and Health (NIOSH), ultrafine titanium dioxide must be considered as a prospective work-related carcinogenic agent, caused by a secondary genotoxicity mechanism common to other poorly soluble or insoluble particles; it is associated with surface area and particle size, and a work-related exposure limit of 0.3 mg/m^3^ must be considered [180]. The aforementioned institution also estimated that there was adequate evidence of CNTs having damaging health effects in humans (respiratory inflammation and fibrosis, and asbestos-type pathology resulting from exposure to straighter, longer carbon nanotube structures), based on animal and in vitro studies [181]. Particulate air pollution is associated with an extensive range of conditions influencing several organs of the human body [182]. Inhaling different nanomaterials at the time of manufacture, molding, coating, as well as inclusion could adversely influence the workers, thus leading to a severe health problem. Therefore, proper monitoring of air quality is needed in the course of manufacturing of nanomaterials in a factory [183]. Furthermore, personal shielding equipment (gloves, masks, etc.) can also be employed for mitigating the possible hazards. Moreover, a periodic medical checkup is highly recommended for workers, due to the fact that the suggested personal shielding equipment might be insufficient in certain cases. Predominantly, in the construction and materials industry, there will be additional issues with regard to the final product presentation. Some of the supplied materials will be in the form of a powder, and therefore, the nanomaterials should be stabilized with different techniques. Several protective and preventive measures were presented with respect to the nanomaterial properties. As an example, Díaz-Soler et al. [184] stated five important steps for the management of nanomaterial exposure in construction areas. Significant developments are required until the nanomaterials are nontoxic for all.

### 5.3. Environmental Implications

With the enhancement in the demands and applications of nanomaterials from formerly unrelated industries, the fabrication and exposure of nanomaterials has increased. Therefore, through transportation, disposal, washing, and erosion of the nanomaterial-enhanced products, these nanomaterials will find their way into ecosystems [185]. The influence of the addition of nanomaterials into land and aqueous environments is presently unclear, with uncertain direct results of nanomaterial exposure.

Solid manufactured nanomaterial wastes from fabrication processes or the demolition and construction activities are transferred to legalized disposal sites. Before their dumping, these wastes most probably undergo a crushing process. Successive landfilling, land farming, and incineration might be the predominant methods for the ecological discharge of manufactured nanomaterials in the construction industry. The current available literature related to engineered nanomaterials incorporated in waste products; the possibility for the discharge of engineered nanomaterials from these products; and their probable fate in waste degradation, landfilling, incineration, and recycling was reviewed and assessed by Part et al. [186]. Manufactured nanomaterials reach the waste stream, and subsequently some nanomaterials will be subjected to incineration as part of their end-of-life treatment. Incineration is an important waste treatment method, with high potential for nanomaterial modifications, and either controlling them successfully or discharging these materials to the surroundings. Incineration has been proven to be a safe and an effective end-of-life treatment for limiting the effects of nanomaterials on the environment, as well as on the human health [187]. The present regulations on incinerator emissions do not precisely address nanomaterials; however, limits on metal and particle emissions might prove effective to a certain extent in reducing the discharge of nanomaterials in the incinerator effluent. Aerosolization of manufactured nanomaterials; wastewater effluents from fabrication processes and construction works; as well as corrosion, abrasion, and adhesive wear of civil/building structures, could also lead to the discharge of nanomaterials into the environment. Evaluating the environmental exposure to manufactured nanomaterials on a long-standing basis is a major challenge due to the analytical restrictions that prevent extensive multiphase observation of their transformation, transportation, and fate in the environment.

One of the significant problems with nanomaterials is the manner in which interactions occur involving materials that have been formerly regarded as harmless. Specially, though silver has been considered safe, studies have proposed that silver nanomaterial exposure in the embryonic stage of zebrafishes could stimulate irregularity in development or even death at levels greater than 0.190 nM [188]. Nonmarine illustrations contribute an indication of problems emerging from ecological nanomaterial interaction, with silver-based nanomaterials disturbing the development of seeds in a variety of plants, leading to the accumulation of silver in shoots, and the biological accumulation in gastropods, rag worms, and green algae [189].

Thus, a greater awareness of the novel products being established will also empower the building/construction industries to maximize the advantages that nanotechnology can contribute. With proper comprehension, there will be a development in the usage of products that were previously accessible but hardly specified; e.g., insulation that is four times as efficient as conventional materials, superior-performance concrete that lessens the requirements for steel reinforcement, or photocatalytic concretes that can decrease airborne contamination. It may also inspire the timely implementation of products that are prospective but not yet completely advanced; e.g., photovoltaic cells that can produce electricity from clear windows, or vanadium-based coatings that can eradicate the requirements for the cleaning of windows on high-rise buildings. Nanomaterials can contribute both risks and advantages. Currently, nanomaterials exist in a few commercial construction products; a couple of them are labeled, while others are not, and there are certainly further materials to develop. It is substantial that the building/construction industries identify sufficient information on the nanosized materials to take safe and smart decisions.

## 6. Concrete Recycling

The recycling of concrete is an important measure adopted for reducing the ecological effects of concrete structures at the end of their life cycle [190,191,192]. In recent times, concrete recycling with nanomaterials has been explored, and it was noted to have improved compressive strength as compared to a standard recycled concrete (RC) [193,194,195]. It was noted that the incorporation of nanomaterials in the recycled standard concrete could offer mechanical properties that were the same as pristine standard concrete [196]. The microstructure, as well as strength of concrete, are enhanced; however, the concrete’s workability will be decreased with the inclusion of nanomaterials. Moreover, it can be noted that the nanomaterial-incorporated RC could achieve a similar compressive strength to pristine standard concrete after 28 days, while the concentration of the nanosilica was 3% by mass.

In a study carried out by Agarwal et al. [197], the team examined the flexural strength, tensile strength, compressive strength, and durability of control concrete (with natural aggregate and recycled aggregate) and concrete samples with nanosilica in the recycled aggregate concrete matrix. It was noted that the concrete mix with 3% nanosilica and 40% recycled aggregate showed the optimum results for durability and strength. Moreover, it supported the decreasing of the emissions of carbon dioxide, which occurred due to the greater quantity of cement use, resulting in ecological challenges such as the greenhouse effect. Nanosilica use can enhance the recycled concrete’s microstructure [198]. In a study by Hosseini et al. [198], it was noted that the inclusion of nanosilica in recycled aggregates could decrease the flowability and workability of pristine concrete separately, although the viscosity of recycled fresh concrete was remarkably increased. With the usage of lower concentrations of nanosilica and increasing its concentration up to 3%, the permeability and mechanical properties of recycled concretes were increased.

Wang et al. [199] fabricated recycled aggregate concrete and nanosilica recycled aggregate concrete, and it was noted that the nanosilica 2% solution concentration and 48 h soaking time demonstrated an improved modification effect on the mechanical behavior of the recycled aggregates. Moreover, the nanosilica-incorporated recycled aggregate showed a remarkable impact on the improvement in the compressive strength of the recycled aggregate concrete cube.

A work by Mukharjee et al. [200] addressed the preparation of advanced construction materials using commercially available nanosilica, as well as recycled aggregates obtained from construction–demolition waste. The results confirmed that an increase in compressive strength was accomplished with the inclusion of nanosilica, along with the restoration of a decrease in the compressive strength of recycled aggregate concrete mixes. In the study carried out by Zheng et al. [201], it was noted that nano-SiO_2_, basalt fiber, and composite addition of nano-SiO_2_, basalt fiber, could effectively enhance the interface structure and durability of recycled concrete.

On the other hand, the existing studies on nanomaterial-incorporated concrete are very limited, and therefore additional studies must be carried out to investigate the impact of nanomaterial-incorporated recycled concrete on its dynamic mechanical properties, and compare it with pristine standard concrete with respect to impact loading [196]. Because of the defects in recycled concrete structures and the complexity in the interface framework, additional studies should be carried out. The microproperties of fiber nanorecycled concrete were mainly examined by employing scanning electron microscopy, X-ray diffraction, and other techniques, and the above-mentioned properties should be researched further. Several studies were performed on the durability and carbonation resistance of recycled concrete; though only very limited research has investigated the development of the microstructure at the time of carbonation.

## 7. Future Research

Different bio-based nanomaterials are also recommended for the lowering the negative impact on the environment and on human health. In a study by Bang et al. [202], a new biomolecule, microbial calcite, was presented as a smart nanomaterial for self-healing concrete. The results confirmed that the overall performance of the concrete was remarkably improved by microbial calcite treatment in simulated concrete cracks and cement mortar beams. Barnat-Hunek et al. [203] also confirmed that nanocellulose in concrete could increase the tensile strength in bending. More studies should be focused on developing appropriate bio-nanomaterials that can be used in concrete. In addition, the nanomaterial manufacturers must properly declare all nanoparticle ingredients in their products. Workers must receive appropriate data and training regarding the nanomaterials and their safe handling, and must be consulted on the preparation, organization, and implications for health and safety in the nanomaterials’ application.

According to the current review, the subsequent potential studies are suggested: (1) optimization of nanosilica-incorporated concrete, and the mathematical modelling of behaviour of concrete, need detailed investigation; (2) scientists should develop a high-performance, high-strength, and lightweight nanosilica-incorporated concrete; (3) it is essential to set up a basic approach of mixing design for this nanomaterial-incorporated concrete; (4) the hardened and fresh properties of nanosilica-incorporated concrete, needed for acoustical and thermal properties, should be evaluated; (5) properly examine the engineering properties such as shrinkage, creep, bond, etc. of nanosilica-incorporated concrete; (6) the optimal amount of superplasticizers for improved workability should be assessed; (7) the ultrasonication method demonstrated a noticeable outcome as regards the CNT dispersion, and hence, the concrete manufacturers should modify their facilities for accommodating the sonication technique. Consequently, the overall expenses must be assessed and compared with the attained advantages of employing the CNTs with ordinary Portland cement products; (8) studies on the mechanical characteristics and fundamental model of RC under dynamic actions is inadequate, and hence research in this area must be carried out; (9) proper assessment of the mechanical characteristics of recycled concrete in complex stress states and multiaxial conditions must be performed; (10) research should also be carried out on the development of the microstructure at the time of carbonation; and (11) studies on the durability and mechanical behavior of RC at higher temperatures must be performed, and a higher-temperature resistance modeling of RC must be put forward.

For developing the commercial-scale production of advanced concrete materials, it is very important to have an effective technique for nanomaterial dispersion that will permit an appropriate and stable dispersion in concrete. Moreover, in the future, a significant challenge will be a decrease in the preparation expenses of the nanomaterials. It is always desired to make construction sector materials economical, energy-efficient, and sustainable. Some researchers are already carrying out studies for analyzing the negative impact of nanomaterials for specific applications [204]; however, more studies should be carried out in different sectors. Furthermore, additional studies must be carried out for the proper determination of the optimum concentration of nanomaterials to be included in concrete for obtaining higher durability and mechanical properties. Innovative products such as functional/smart self-healing concrete must have capability of delivering the traditional materials, such as handling the released admixture for penetrating into the market.

## 8. Discussion and Conclusions

In recent times, advanced nanotechnology has been meritoriously employed in various sectors for the comfort and welfare of humanity. Conversely, any groundbreaking unendorsed technology will come with some disadvantages. Presently, there are certain doubts regarding the conceivable unsafe influences of the nanomaterials on the surroundings. In general, the advanced nanomaterial-containing concrete for sustainable progress, being the century’s main priority, have prompted significant financial investment in the studies for defining novel avenues in the concrete field, suggesting the possibility of reducing ecological contaminations. A majority of the nanostructure-based products applied to date in the concrete sector have numerous advantages. There are several explanations for accepting the fact that the nanomaterial usage is rising. Sometimes, the nanomaterial production might need comparatively higher energy. Innovative concrete structures need structural components with superior mechanical properties, as well as higher durability. The best solution is the incorporation of nanostructured materials in concrete mixes, which could improve the mechanical properties. Nanomaterials such as nanosilica, nano-TiO2, CNTs, etc. have the capability for increasing the durability of buildings by improving mechanical and thermal properties, which could result in an indirect reduction in energy usage and total expenses in the concrete industry. The microstructures of various kinds of concrete is remarkably improved by the inclusion of nanomaterials, as the nanomaterials enhance the hydration process by generating more hydrated products. Incorporating various nanomaterials into the concrete mixes decreased the setting time as well as workability, which was due to the stronger reactivity of nanomaterials having a larger surface-to-volume ratio. Improvement in compressive strength could be observed with an improvement in the replacement ratio of nanomaterials. This was due to the improved hydration of a more compacted microstructure with the incorporation of finely dispersed nanomaterials. Moreover, it was noted that the nanomaterials could increase the concrete beam ductility by approximately 150%. With the incorporation of a nanomaterial such as CNT, the concrete compressive strength could be improved to 21%, and also it can result in a decrease in the early as well as long-term shrinking of concrete of 54.0% and 15.0%, respectively. Moreover, the CNT addition to concrete showed 17.0% to 18.0% reduced long-term creep as compared to the concrete with no CNTs. The inclusion of nano-titanium oxide in cement matrices could offer concrete frameworks with self-sensing as well as self-cleaning capabilities. Overall, it was noted that optimum concentrations of nanomaterial incorporation in the concrete could enhance its flexural, tensile, and compressive strength, along with the workability and water absorption. High-performance nanomaterial-incorporated concretes could positively impact the increase in the sustainability and durability of the constructions. The self-healing sustainable concrete frameworks obtained from different nanomaterials could restore the weakened constructions in an advanced manner at lesser expense, relative to that accomplished by epoxy-containing healing products, therefore attaining the ecological sustainability. The application of specific nanostructured materials could improve the performance of concrete, as well as its life cycle. The nanomaterial-incorporated concrete could permit the development of ultra-strength concrete frameworks having higher durability, and thereby decreasing the maintenance needs.

It is extremely important to develop an advanced procedure to make sure the appropriate nanomaterial dispersion commercial-scale field applications. Effective dispersion of nanoparticles is key to achieving the full benefits of adding nanoparticles in cementitious system. So far, the nano-based technology is helping significantly for developing many technologies along with industrial sectors: including the construction sector. Because of the nonexistence of disposal guidelines, the amount of nanosized materials present in the surroundings is continuously increasing. Nano-based concrete structures were established to be ecofriendly with respect to greenhouse gas emissions, energy storage, and sustainable development. Thus, the current, as well as forthcoming, trends are to substitute the conventional ordinary Portland cement-based concretes with functional/smart nano-based concrete structures for sustainable development

It was noted the nanomaterials can play an important role in the development of construction materials, and thus proper understanding of these nanomaterials is extremely important. Along with the substantial advancement of nanomaterials in different applications, it should also be noted that the nanomaterials are still in their developing stages, and they definitely have many challenges and unsolved complications for their huge commercial prospects. The experimental test results of nano-reinforced cement specimens have confirmed that they could improve the durability and mechanical strength of the resultant concretes. This was also confirmed by different studies performed by scientists on the nano-reinforced cement [204,205]. The building material and construction sector have added difficulties due to its huge scale and also because of the current manufacturing procedures that possibly need to be completely redesigned when the nanomaterials are being used. Human exposure to nanomaterials from construction materials is increasing; conversely, regrettably this is not complemented by the proper limits or/and awareness recommended by safety regulations with respect to their toxicology data. It could be predicted that this situation may change soon. There must be a distinct administration body with a guideline that notifies the manufacturers about the safer limits for their specific safety and the public safety. A rise in the concentration of nanomaterial in the advanced smart nano-based concrete structures greater than the optimum amount was demonstrated to show adverse impact on the strength performance and durability, which was mostly because of the less compaction, nonuniform dispersion, and development of weaker sections in the concrete. Modern science, predominantly the analytic chemistry with the advancements in instrument analytic techniques, is offering a wide spectrum of many methods that can be used to monitor nanomaterials present in the construction material containing wastewater. Research studies should be performed to prevent the probable human health dangers for the users, including the adult population, then again specifically for the children and for the babies.

Major conclusions from this study are: (i) the construction sector has higher prospects of nanotechnology for solving certain complicated problems existing in this area; (ii) optimum addition of appropriate nanomaterial to the concrete can improve the mechanical properties such as tensile strength, compressive strength, flexural strength, durability, etc. of the concrete; (iii) freeze–thaw resistance and abrasion resistance of the concrete can also be improved with the addition of nanosilica to the concrete mix; (iv) nanomaterials can offer concrete structures with self-sensing and self-cleaning abilities; (v) however, the concentration of the nanomaterial present in the concrete should be in a very limited quantity; (v) the lack of information on nanomaterial toxicology topics and a tendency to disconnect from prevailing research and development is a significant blockade for its development; (vi) to suppress these type of restrictions, organized construction sector-strategic policies should be followed to benefit from the nanomaterials to attain a reasonable benefit in particular application extents and the product categories.

This study offered a complete and detailed analysis of improvement in concrete’s properties with the addition of nanomaterials, its risk evaluation, related health issues, and environmental implications of nanomaterial-based materials for the application in concrete sector. The review focused on the environmental impact and the future scope of the nano-based concrete materials. It was clearly noted that the nanostructured materials have higher capability in smart infrastructure applications with superior strength concrete frameworks. This study also featured a discussion on the prevailing issues and barriers to the commercialization of nano-based construction materials that harness the benefits of nanoparticles. It can be concluded that the application of nanomaterials in the construction sector can offer both risks and benefits. Presently, the nanomaterials are existing in some specific commercial items, and some of these are labeled, whereas some are not labeled. Hence, it is important that the construction sector must have satisfactory knowledge on the nanomaterials, and this will enable them to take safer selections. The main findings from this study are expected to raise awareness on the probable effect of construction wastes with nanomaterials in it and the importance of proper regulations regarding the final disposal of these construction wastes. As the production of concrete is accountable for higher than 5.0% of total emission of CO_2_ globally, the addition of nanomaterials into the concrete mix reduces the essential size of concrete structures, leading to a lower emission of CO_2_ thereby making concrete an eco-friendly construction material. Thus, the environment pollution could remarkably decreased by applying high strong cement-based composites manufactured utilizing different nanostructured materials. Consequently, in the area of construction, the manufacture of nano-based materials is going to perform a substantial part toward sustainable development in the coming period. The application of nano-based concrete materials is beneficial with respect to enhanced engineering properties of cement materials, particularly for the production of sustainable as well as self-healing concretes.

## Figures and Tables

**Table 1:** Some of the utilizations of nanomaterials in the construction industry.

Sl. No.	Nanomaterial Used	Application Area in Construction	Properties	Reference
1	Aluminium oxide nanoparticles	Asphalt concrete, timber	Increased serviceability	[39]
2	Carbon nanotubes	Concrete	Crack prevention, mechanical durability	[40]
3	Titania nanoparticles	Concrete	Self-cleaning, increased degree of hydration	[41]
4	Silica nanoparticles	Concrete	Rapid hydration, reinforcement of mechanical strength	[42]
5	Copper nanoparticles	Steel	Formability, corrosion resistance	[43]
6	Iron oxide nanoparticles	Concrete	Abrasion-resistant, increased compressive strength	[44]
7	Clay nanoparticles	Bricks and mortar	Increased surface roughness and compressive strength	[45]

**Table 2:** Nanomaterial applications in concrete.

Sl. No.	Nanomaterial Used	Function	Details	Reference
1	Nanosilica	Overcome physical deteriorations—shrinkage	Nanosilica aggravated the drying shrinkage when admixed in cement composites	[109]
2	Carbon nanotubes	Overcome physical deteriorations—shrinkage	The admixed CNT decreased the porosity of the cementitious matrix	[110]
3	Nanosilica	Overcome physical deteriorations—freeze–thaw damage	Nanosilica reduced the frost damage	[111]
4	Nanosilica	Overcome physical deteriorations—freeze–thaw damage	Nanosilica improved the freeze–thaw resistance	[112]
5	Nanosilica	Overcome physical deteriorations—freeze–abrasion/erosion	Nanosilica increased the abrasion resistance	[109]
6	Nano-TiO_2_	Overcome physical deteriorations—freeze–abrasion/erosion	Nano-TiO2 increased the abrasion resistance	[113]
7	Nanosilica	Overcome chemical deteriorations—sulfate attack	Nanosilica decreased the mortar expansion	[114]
8	Nanosilica	Overcome chemical deteriorations—thermal degradation	Nanosilica increased the thermal resistance of the cement mortar	[115]
9	Carbon nanotubes and nanosilica	Improvement of mechanical properties	Compressive and flexural strength were enhanced for the nano-admixed mortar	[116]
10	Carbon nanotubes and nanosilica	Improvement of mechanical properties	Nanomaterials improved the compressive strength	[117]

**Table 3:** Overview of the improvements in concrete properties with the addition of nanomaterials.

Sl. No.	Nanomaterials Used	Property Improvements in Concrete
1	Nanosilica	Contributes to reduced emissions of CO_2_, as the addition of 1 kg microsilica reduced almost 4 kg cement, and this can be higher if nanosilica is used Offers increased durability to concrete Improves compressive strength Increases the flexural strength, Improves the tensile strength
2	Carbon nanotubes	Decreases the concrete’s final setting time, Restricts crack development and propagation at early ages, Produces dense concrete, Increased quality of bond interaction between aggregates and cement paste Increases the compressive strength Improves the tensile strength, Young’s modulus, flexural strength, and fracture toughness Decreases the required size of concrete structural members Decreases the early and long-term shrinkage of concrete
3	Nano-TiO_2_	Increases the abrasion resistance of concrete Improves compressive strength Increases the durability of concrete structures Increases the flexural strength Speeds up the early-age hydration of ordinary Portland cement Offers self-sensing and self-cleaning properties to concrete structures
4	Nano-Fe_2_O_3_	Improves compressive strength Increases the flexural strength Improves split tensile strength Reduces the setting time of fresh concrete Decreases the total porosity of concrete Improves concrete’s abrasion resistance

**Table 4:** Some nanoparticles used in the construction industry that are toxic to human organs.

Sl. No.	Nanomaterial	Construction Industry Application	Affected System/Organ/Cell	References
1	Carbon nanotubes	Concrete, ceramics	Inflammation, oxidative stress	[173]
2	SiO_2_ nanoparticles	Windows, ceramics, concrete	Bronchoalveolar carcinoma-derived cells	[174]
3	TiO_2_ nanoparticles	Windows, cement	Cell death, carcinogenesis, metabolic changes	[175]
4	Silver nanoparticles	Biocidal activity	Fibroblast, reproductive organs, vascular system, carcinogenesis, brain, liver, lungs, immune system	[176]
5	Iron oxide nanoparticles	Concrete	Oxidative DNA damage	[177]
6	Zinc oxide Nanoparticles	Sensors	Cell proliferation	[178]

## Data Availability

Data sharing is not applicable.

## References

[1] He X., Deng H., Hwang H.M. (2019). The current application of nanotechnology in food and agriculture. J. Food Drug Anal..

[2] Svendsen C., Walker L.A., Matzke M., Lahive E., Harrison S., Crossley A.A., Park B., Lofts S., Lynch I., Vázquez-Campos S. (2020). Key principles and operational practices for improved nanotechnology environmental exposure assessment. Nat. Nanotechnol..

[3] Sanzari I., Leone A., Ambrosone A. (2019). Nanotechnology in plant science: To make a long story short. Front. Bioeng. Biotechnol..

[4] Saleem H., Trabzon L., Kilic A., Zaidi S.J. (2020). Recent advances in nanofibrous membranes: Production and applications in water treatment and desalination. Desalination.

[5] Yadav S., Saleem H., Ibrar I., Naji O., Hawari A.A., Alanezi A.A., Zaidi S.J., Altaee A., Zhou J. (2020). Recent developments in forward osmosis membranes using carbon-based nanomaterials. Desalination.

[6] Saleem H., Zaidi S.J. (2020). Nanoparticles in reverse osmosis membranes for desalination: A state of the art review. Desalination.

[7] Guisbiers G., Mejía-Rosales S., Deepak F.L. (2012). Nanomaterial properties: Size and shape dependencies. J. Nanomater..

[8] Yaqoob A.A., Parveen T., Umar K., Mohamad Ibrahim M.N. (2020). Role of nanomaterials in the treatment of wastewater: A review. Water.

[9] Saleem H., Zaidi S.J. (2020). Sustainable Use of Nanomaterials in Textiles and Their Environmental Impact. Materials.

[10] Malhotra B.D., Ali M.A. (2018). Nanomaterials in Biosensors. Nanomater. Biosens..

[11] Zaidi S.J., Fadhillah F., Saleem H., Hawari A., Benamor A. (2019). Organically modified nanoclay filled thin-film nanocomposite membranes for reverse osmosis application. Materials.

[12] Saleem H., Zaidi S.J. (2020). Recent Developments in the Application of Nanomaterials in Agroecosystems. Nanomaterials.

[13] Saleem H., Zaidi S.J. (2020). Developments in the Application of Nanomaterials for Water Treatment and Their Impact on the Environment. Nanomaterials.

[14] Saleem H., Javaid Zaidi S. (2020). Innovative Nanostructured Membranes for Reverse Osmosis Water Desalination, Qatar University Annual Research Forum Exhibition, QUARFE 2020 Theme 1: Energy. https://qspace.qu.edu.qa/handle/10576/16558.

[15] Khare S., Williams K., Gokulan K. (2014). Nanotechnology. Encyclopedia of Food Microbiology.

[16] Almeida L., Felzenszwalb I., Marques M., Cruz C. (2020). Nanotechnology activities: Environmental protection regulatory issues data. Heliyon.

[17] Kamarulzaman N.A., Lee K.E., Siow K.S., Mokhtar M. (2020). Public benefit and risk perceptions of nanotechnology development: Psychological and sociological aspects. Technol. Soc..

[18] Anandharamakrishnan C. (2020). Trends and Impact of Nanotechnology in Agro-Food Sector. Ref. Modul. Food Sci..

[19] Rodriguez-Narvaez O.M., Peralta-Hernandez J.M., Goonetilleke A., Bandala E.R. (2019). Biochar-supported nanomaterials for environmental applications. J. Ind. Eng. Chem..

[20] Gong C., Sun S., Zhang Y., Sun L., Su Z., Wu A., Wei G. (2019). Hierarchical nanomaterials via biomolecular self-assembly and bioinspiration for energy and environmental applications. Nanoscale.

[21] Singh B., Na J., Konarova M., Wakihara T., Yamauchi Y., Salomon C., Gawande M.B. (2020). Functional Mesoporous Silica Nanomaterials for Catalysis and Environmental Applications. Bull. Chem. Soc. Jpn..

[22] Yang L., Yang L., Ding L., Deng F., Luo X.B., Luo S.L. (2019). Principles for the Application of Nanomaterials in Environmental Pollution Control and Resource Reutilization. Nanomaterials for the Removal of Pollutants and Resource Reutilization.

[23] Zhang H., Zhu C., Wei C., Duan H., Yu J. (2020). Application of functionalized nanomaterials in asphalt road construction materials. Handbook of Functionalized Nanomaterials for Industrial Applications.

[24] Díaz-Soler B., López-Alonso M., Martínez-Aires M.D. (2019). Nanoenabled Products Applied on Construction Sector. New Risks for Workers. International Congress on Engineering and Sustainability in the XXI Century.

[25] Díaz-Soler B.M., Martínez-Aires M.D., López-Alonso M. (2019). Potential risks posed by the use of nano-enabled construction products: A perspective from coordinators for safety and health matters. J. Clean. Prod..

[26] Tanzadeh J. (2020). Laboratory evaluation of self-compacting fiber-reinforced concrete modified with hybrid of nanomaterials. Constr. Build. Mater..

[27] Ram V.V., Singhal R., Parameshwaran R. (2020). Energy efficient pumpable cement concrete with nanomaterials embedded PCM for passive cooling application in buildings. Mater. Today Proc..

[28] Singh N.B. (2020). Properties of cement and concrete in presence of nanomaterials. Smart Nanoconcretes and Cement-Based Materials.

[29] Pacheco-Torgal F. (2019). Introduction to nanotechnology in eco-efficient construction. Nanotechnology in Eco-Efficient Construction.

[30] Shah K.W., Li W. (2019). A review on catalytic nanomaterials for volatile organic compounds VOC removal and their applications for healthy buildings. Nanomaterials.

[31] Dahlan A.S. (2019). Smart and Functional Materials Based Nanomaterials in Construction Styles in Nano-Architecture. Silicon.

[32] Aljenbaz A.Z., Çağnan Ç. (2020). Evaluation of Nanomaterials for Building Production within the Context of Sustainability. Eur. J. Sustain. Dev..

[33] Huseien G.F., Shah K.W., Sam A.R.M. (2019). Sustainability of nanomaterials based self-healing concrete: An all-inclusive insight. J. Build. Eng..

[34] Pacheco-Torgal F., Jalali S. (2011). Nanotechnology: Advantages and drawbacks in the field of construction and building materials. Constr. Build. Mater..

[35] Solano R., Patiño-Ruiz D., Herrera A. (2020). Preparation of modified paints with nano-structured additives and its potential applications. Nanomater. Nanotechnol..

[36] Zheng L., Xiong T., Shah K.W. (2019). Transparent nanomaterial-based solar cool coatings: Synthesis, morphologies and applications. Sol. Energy.

[37] Vidales-Herrera J., López I. (2020). Nanomaterials in coatings: An industrial point of view. Handbook of Nanomaterials for Manufacturing Applications.

[38] Saleem H., Zaidi S.J., Ismail A.F., Goh P.S. (2022). Advances of nanomaterials for air pollution remediation and their impacts on the environment. Chemosphere.

[39] Ali S.I.A., Amiruddin I., Nur I.M.Y., Norhidayah A.H., Ahmad N.H.I. (2016). Characterization of the performance of aluminum oxide nanoparticles modified asphalt binder. J. Teknol..

[40] Hassan A., Hala E., Ibrahim G.S. (2019). Effect of Adding Carbon Nanotubes on Corrosion Rates and Steel-Concrete Bond. Sci. Rep..

[41] Yu X., Shaobo K., Xu L. (2018). Compressive strength of concrete reinforced by TiO2 nanoparticles. AIP Conf. Proc..

[42] Palla R., Karade S.R., Mishra G., Sharma U., Singh L.P. (2017). High strength sustainable concrete using silica nanoparticles. Constr. Build. Mater..

[43] Mohajerani A., Burnett L., Smith J.V., Kurmus H., Milas J., Arulrajah A., Horpibulsuk S., Kadir A.A. (2019). Nanoparticles in construction materials and other applications, and implications of nanoparticle use. Materials.

[44] Horszczaruk E. (2019). Properties of cement-based composites modified with magnetite nanoparticles: A review. Materials.

[45] Karozou A., Eleni P., Stefanidou M. (2019). Enhancing Properties of Clay Mortars Using Nano-Additives. Solid State Phenom..

[46] Brooks J. (2003). Elasticity, shrinkage, creep and thermal movement. Advanced Concrete Technology.

[47] Aïtcin P.C. (2016). Portland cement. Science and Technology of Concrete Admixtures.

[48] Leone M.F. (2012). Nanotechnology for architecture. Innovation and eco-efficiency of nanostructured cement-based materials. J. Arch. Eng. Technol..

[49] How Cement is Made? Portland Cement Association. https://www.cement.org/cement-concrete/how-cement-is-made.

[50] Ghasemzadeh F., Manafpour A., Sajedi S., Shekarchi M., Hatami M. (2016). Predicting long-term compressive creep of concrete using inverse analysis method. Constr. Build. Mater..

[51] Aili A., Vandamme M., Torrenti J.M., Masson B. (2018). Is long-term autogenous shrinkage a creep phenomenon induced by capillary effects due to self-desiccation?. Cem. Concr. Res..

[52] Claisse P. (2016). Introduction to cement and concrete. Civil Engineering Materials.

[53] Wang Y.L., Lee Y.H., Chiu I.J., Lin Y.F., Chiu H.W. (2020). Potent impact of plastic nanomaterials and micromaterials on the food chain and human health. Int. J. Mol. Sci..

[54] Hochella M.F., Mogk D.W., Ranville J., Allen I.C., Luther G.W., Marr L.C., Sahai N., McGrail B.P., Murayama M., Qafoku N.P. (2019). Natural, incidental, and engineered nanomaterials and their impacts on the Earth system. Science.

[55] Miernicki M., Hofmann T., Eisenberger I., von der Kammer F., Praetorius A. (2019). Legal and practical challenges in classifying nanomaterials according to regulatory definitions. Nat. Nanotechnol..

[56] Demir E. (2020). A review on nanotoxicity and nanogenotoxicity of different shapes of nanomaterials. J. Appl. Toxicol..

[57] Yuvaraj M., Yuvaraj V., Arunkumar V., Pandiyan M., Subramanian K.S. (2020). Nanosafety. Biochemical Toxicology-Heavy Metals and Nanomaterials.

[58] Riediker M. (2020). Nano-safety research lessons for dealing with aerosol transmissions of COVID-19. Nanotoxicology.

[59] Cronin J.G., Jones N., Thornton C.A., Jenkins G.J., Doak S.H., Clift M.J. (2020). Nanomaterials and innate immunity: A perspective of the current status in nanosafety. Chem. Res. Toxicol..

[60] Winkler D.A. (2020). Role of Artificial Intelligence and Machine Learning in Nanosafety. Small.

[61] Trybula W., Newberry D. (2020). Progress and Upcoming Challenges of Nano-Safety: Education, Manufacture, and Long-Term Impacts. IEEE Nanotechnol. Mag..

[62] Valdiglesias V., Laffon B. (2020). The impact of nanotechnology in the current universal COVID-19 crisis. Let’s not forget nanosafety!. Nanotoxicology.

[63] Wigger H., Nowack B. (2019). Material-specific properties applied to an environmental risk assessment of engineered nanomaterials—Implications on grouping and read-across concepts. Nanotoxicology.

[64] Isigonis P., Afantitis A., Antunes D., Bartonova A., Beitollahi A., Bohmer N., Doak S., Bouman E., Chaudhry Q., Cimpan M.R. (2020). Risk Governance of Emerging Technologies Demonstrated in Terms of its Applicability to Nanomaterials. Small.

[65] Kühnel D., Nickel C., Hellack B., van der Zalm E., Kussatz C., Herrchen M., Meisterjahn B., Hund-Rinke K. (2019). Closing gaps for environmental risk screening of engineered nanomaterials. NanoImpact.

[66] Auffan M., Masion A., Mouneyrac C., de Garidel-Thoron C., Hendren C.O., Thiery A., Santaella C., Giamberini L., Bottero J.Y., Wiesner M.R. (2019). Contribution of mesocosm testing to a single-step and exposure-driven environmental risk assessment of engineered nanomaterials. NanoImpact.

[67] Crucho J.M.L., das Neves J.M.C., Capitão S.D., de Picado-Santos L.G. (2019). Evaluation of the durability of asphalt concrete modified with nanomaterials using the TEAGE aging method. Constr. Build. Mater..

[68] Singh N.B., Saxena S.K., Kumar M. (2018). Effect of nanomaterials on the properties of geopolymer mortars and concrete. Mater. Today Proc..

[69] Huguet-Casquero A., Gainza E., Pedraz J.L. (2020). Towards green nanoscience: From extraction to nanoformulation. Biotechnol. Adv..

[70] Yoonus J., Resmi R., Beena B. (2020). Greener nanoscience: Piper betel leaf extract mediated synthesis of CaO nanoparticles and evaluation of its antibacterial and anticancer activity. Mater. Today Proc..

[71] Zhuang C., Chen Y. (2019). The effect of nano-SiO2 on concrete properties: A review. Nanotechnol. Rev..

[72] Cai X., Hong R.Y., Wang L.S., Wang X.Y., Li H.Z., Zheng Y., Wei D.G. (2009). Synthesis of silica powders by pressured carbonation. Chem. Eng. J..

[73] Hong R.Y., Feng B., Ren Z.Q., Xu B., Li H.Z., Zheng Y., Ding J., Wei D.G. (2009). Thermodynamic, hydrodynamic, particle dynamic, and experimental analyses of silica nanoparticles synthesis in diffusion flame. Can. J. Chem. Eng..

[74] Hong R., Ding J., Li H. (2003). Thermodynamic analysis and experimental verification for synthesizing silicon nitride nanoparticles using RF plasma CVD. China Particuology.

[75] Al-Abboodi S.M.T., Al-Shaibani E.J.A., Alrubai E.A. (2020). Preparation and Characterization of Nano silica Prepared by Different Precipitation Methods. IOP Conf. Ser. Mater. Sci. Eng..

[76] Che J., Cagin T., Goddard W.A. (2000). Thermal conductivity of carbon nanotubes. Nanotechnology.

[77] Pandey P., Dahiya M. (2016). Carbon nanotubes: Types, methods of preparation and applications. Carbon.

[78] Macak J. (2008). Growth of Anodic Self-Organized Titanium Dioxide Nanotube Layers. https://opus4.kobv.de/opus4-fau/frontdoor/index/index/docId/634.

[79] Pratsinis S.E., Ensor D.S. (2011). History of Manufacture of Fine Particles in High-Temperature Aerosol Reactors. Aerosol Science and Technology: History and Reviews.

[80] Chen X., Mao S.S. (2007). Titanium dioxide nanomaterials: Synthesis, properties, modifications, and applications. Chem. Rev..

[81] Joshaghani A., Balapour M., Mashhadian M., Ozbakkaloglu T. (2020). Effects of nano-TiO_2_, nano-Al_2_O_3_, and nano-Fe_2_O_3_ on rheology, mechanical and durability properties of self-consolidating concrete (SCC): An experimental study. Constr. Build. Mater..

[82] Piqué T.M., Balzamo H., Vázquez A. (2011). Evaluation of the hydration of portland cement modified with polyvinyl alcohol and nano clay. Key Eng. Mater..

[83] Norhasri M.M., Hamidah M.S., Fadzil A.M. (2017). Applications of using nano material in concrete: A review. Constr. Build. Mater..

[84] AlZainati N., Saleem H., Altaee A., Zaidi S.J., Mohsen M., Hawari A., Millar G.J. (2021). Pressure retarded osmosis: Advancement, challenges and potential. J. Water Process. Eng..

[85] Elrahman M.A., Chung S.-Y., Sikora P., Rucinska T., Stephan D. (2019). Influence of nanosilica on mechanical properties, sorptivity, and microstructure of lightweight concrete. Materials.

[86] Rollins A.B., Collet P.E.P., Andres V. Concrete Porosity Reduction by Colloidal Silica Nano Technology, Part 2: One Year Results from Djeno Wharf. https://www.pci.org/PCI_Docs/Papers/2018/10001_Final_Paper.pdf.

[87] Mostafa S.A., Faried A.S., Farghali A.A., El-Deeb M.M., Tawfik T.A., Majer S., Elrahman M.A. (2020). Influence of nanoparticles from waste materials on mechanical properties, durability and microstructure of UHPC. Materials.

[88] Singh L.P., Karade S.R., Bhattacharyya S.K., Yousuf M.M., Ahalawat S. (2013). Beneficial role of nanosilica in cement based materials—A review. Constr. Build. Mater..

[89] Li W., Huang Z., Cao F., Sun Z., Shah S.P. (2015). Effects of nano-silica and nano-limestone on flowability and mechanical properties of ultra-high-performance concrete matrix. Constr. Build. Mater..

[90] Najigivi A., Khaloo A., Rashid S.A. (2013). Investigating the effects of using different types of SiO2 nanoparticles on the mechanical properties of binary blended concrete. Compos. Part B Eng..

[91] Barbhuiya G.H., Moiz M.A., Hasan S.D., Zaheer M.M. (2020). Effects of the nanosilica addition on cement concrete: A review. Mater. Today Proc..

[92] Sobolev K., Gutiérrez M.F. (2005). How nanotechnology can change the concrete world: Part two of a two-part series. Am. Ceram. Soc. Bull..

[93] Kim G.M., Yang B.J., Ryu G.U., Lee H.K. (2016). The electrically conductive carbon nanotube (CNT)/cement composites for accelerated curing and thermal cracking reduction. Compos. Struct..

[94] Mohsen M.O., Al Ansari M.S., Taha R., Al Nuaimi N., Taqa A.A. (2019). Carbon nanotube effect on the ductility, flexural strength, and permeability of concrete. J. Nanomater..

[95] Yıldırım G., Sarwary M.H., Al-Dahawi A., Öztürk O., Anıl Ö., Şahmaran M. (2018). Piezoresistive behavior of CF- and CNT-based reinforced concrete beams subjected to static flexural loading: Shear failure investigation. Constr. Build. Mater..

[96] Adhikary S.K., Rudzionis Z., Ghosh R. (2021). Influence of CNT, graphene nanoplate and CNT-graphene nanoplate hybrid on the properties of lightweight concrete. Mater. Today Proc..

[97] Lushnikova A., Zaoui A. (2017). Improving mechanical properties of CSH from inserted carbon nanotubes. J. Phys. Chem. Solids.

[98] Chandran R.B. (2020). Macroscopic CNTs as promising reinforcements for concrete structures. Smart Nanoconcretes and Cement-Based Materials.

[99] Yu Z., Lau D. (2017). Evaluation on mechanical enhancement and fire resistance of carbon nanotube (CNT) reinforced concrete. Coupled Syst. Mech..

[100] Jung M., Lee Y.S., Hong S.G., Moon J. (2020). Carbon nanotubes (CNTs) in ultra-high performance concrete (UHPC): Dispersion, mechanical properties, and electromagnetic interference (EMI) shielding effectiveness (SE). Cem. Concr. Res..

[101] Hawreen A., Bogas J.A. (2019). Creep, shrinkage and mechanical properties of concrete reinforced with different types of carbon nanotubes. Constr. Build. Mater..

[102] Zanfir A.V., Voicu G., Bădănoiu A.I., Gogan D., Oprea O., Vasile E. (2018). Synthesis and characterization of titania-silica fume composites and their influence on the strength of self-cleaning mortar. Compos. Part B Eng..

[103] Nikbin I.M., Mehdipour S., Dezhampanah S., Mohammadi R., Mohebbi R., Moghadam H.H., Sadrmomtazi A. (2020). Effect of high temperature on mechanical and gamma ray shielding properties of concrete containing nano-TiO_2_. Radiat. Phys. Chem..

[104] Cardellicchio L. (2018). On conservation issues of contemporary architecture: The technical design development and the ageing process of the Jubilee Church in Rome by Richard Meier. Front. Arch. Res..

[105] Kani E.N., Rafiean A.H., Alishah A., Astani S.H., Ghaffar S.H. (2021). The effects of Nano-Fe2O3 on the mechanical, physical and microstructure of cementitious composites. Constr. Build. Mater..

[106] Heikal M., Zaki M.E., Ibrahim S.M. (2021). Characterization, hydration, durability of nano-Fe2O3-composite cements subjected to sulphates and chlorides media. Constr. Build. Mater..

[107] Plank J., Sakai E., Miao C.W., Yu C., Hong J.X. (2015). Chemical admixtures—Chemistry, applications and their impact on concrete microstructure and durability. Cem. Concr. Res..

[108] Barnat-Hunek D., Szymańska-Chargot M., Jarosz-Hadam M., Łagód G. (2019). Effect of cellulose nanofibrils and nanocrystals on physical properties of concrete. Constr. Build. Mater..

[109] Gao Y., He B., Li Y., Tang J., Qu L. (2017). Effects of nano-particles on improvement in wear resistance and drying shrinkage of road fly ash concrete. Constr. Build. Mater..

[110] Konsta-Gdoutos M.S., Metaxa Z.S., Shah S.P. (2010). Multi-scale mechanical and fracture characteristics and early-age strain capacity of high performance carbon nanotube/cement nanocomposites. Cem. Concr. Compos..

[111] Gonzalez M., Tighe S.L., Hui K., Rahman S., Lima A.D.O. (2016). Evaluation of freeze/thaw and scaling response of nanoconcrete for Portland Cement Concrete (PCC) pavements. Constr. Build. Mater..

[112] Quercia G., Spiesz P., Hüsken G., Brouwers J. (2012). Effects of amorphous nano-silica additions on mechanical and durability performance of SCC mixtures. Proceedings of the International Congress on Durability of Concrete (ICDC 2012), Trondhelm, Norway, 18–21 June 2012.

[113] Li H., Zhang M.H., Ou J.P. (2006). Abrasion resistance of concrete containing nano-particles for pavement. Wear.

[114] Ghafoori N., Najimi M. (2016). Sulfate resistance of nanosilica and microsilica contained mortars. ACI Mater. J..

[115] Horszczaruk E., Sikora P., Cendrowski K., Mijowska E. (2017). The effect of elevated temperature on the properties of cement mortars containing nanosilica and heavyweight aggregates. Constr. Build. Mater..

[116] Zaheer M.M., Hasan S.D. (2021). Mechanical and durability performance of carbon nanotubes (CNTs) and nanosilica (NS) admixed cement mortar. Mater. Today Proc..

[117] Narasimman K., Jassam T.M., Velayutham T.S., Yaseer M.M.M., Ruzaimah R. (2020). The synergic influence of carbon nanotube and nanosilica on the compressive strength of lightweight concrete. J. Build. Eng..

[118] De Volder M.F., Tawfick S.H., Baughman R.H., Hart A.J. (2013). Carbon nanotubes: Present and future commercial applications. Science.

[119] World’s First Super-Growth Carbon Nanotube Mass Production PlantOpens, ZEON, Press Releases. https://www.zeon.co.jp/en/news/assets/pdf/151104.pdf.

[120] Zhao W., Yu X., Peng S., Luo Y., Li J., Lu L. (2021). Construction of nanomaterials as contrast agents or probes for glioma imaging. J. Nanobiotechnol..

[121] Vinodhini C., Rajeshkumar V., Anandraj S., Kavitha R., Logsehwaran S., Kapildev C. (2021). Review of Nanomaterials in Construction. IOP Conf. Ser. Mater. Sci. Eng..

[122] Nawar A.H. (2021). Nano-technologies and nano-materials for civil engineering construction works applications. Mater. Today Proc..

[123] Ou C., Wang D. (2021). Structural Performance Characteristics of Nanomaterials and Its Application in Traditional Architectural Cultural Design and Landscape Planning. Adv. Civ. Eng..

[124] Verma A., Yadav M. (2021). Application of nanomaterials in architecture—An overview. Mater. Today Proc..

[125] Ashish P.K., Singh D. (2021). Use of nanomaterial for asphalt binder and mixtures: A comprehensive review on development, prospect, and challenges. Road Mater. Pavement Des..

[126] Sanchez F., Sobolev K. (2010). Nanotechnology in concrete—A review. Constr. Build. Mater..

[127] Colangiuli D., Calia A., Bianco N. (2015). Novel multifunctional coatings with photocatalytic and hydrophobic properties for the preservation of the stone building heritage. Constr. Build. Mater..

[128] Papanikolaou I., de Souza L.R., Litina C., Al-Tabbaa A. (2021). Investigation of the dispersion of multi-layer graphene nanoplatelets in cement composites using different superplasticiser treatments. Constr. Build. Mater..

[129] Chanda S., Bajwa D.S. (2021). A review of current physical techniques for dispersion of cellulose nanomaterials in polymer matrices. Rev. Adv. Mater. Sci..

[130] Mudimela P.R., Nasibulina L.I., Nasibulin A.G., Cwirzen A., Valkeapää M., Habermehl-Cwirzen K., Malm J.E., Karppinen M.J., Penttala V., Koltsova T.S. (2009). Synthesis of carbon nanotubes and nanofibers on silica and cement matrix materials. J. Nanomater..

[131] Wu S., Tahri O. (2021). State-of-art carbon and graphene family nanomaterials for asphalt modification. Road Mater. Pavement Des..

[132] Ali R.A., Kharofa O.H. (2021). The impact of nanomaterials on sustainable architectural applications smart concrete as a model. Mater. Today Proc..

[133] Feng H., Zhao X., Li L., Zhao X., Gao D. (2021). Water stability of bonding properties between nano-Fe2O3-modified magnesium-phosphate-cement mortar and steel fibre. Constr. Build. Mater..

[134] Hou L., Li J., Lu Z., Niu Y. (2021). Influence of foaming agent on cement and foam concrete. Constr. Build. Mater..

[135] Srinivas K. (2014). Nanomaterials for concrete technology. Int. J. Civ. Struct. Environ. Infrastruct. Eng. Res. Dev..

[136] Gamal H.A., El-Feky M.S., Alharbi Y.R., Abadel A.A., Kohail M. (2021). Enhancement of the concrete durability with hybrid nano materials. Sustainability.

[137] Onaizi A.M., Huseien G.F., Lim N.H.A.S., Amran M., Samadi M. (2021). Effect of nanomaterials inclusion on sustainability of cement-based concretes: A comprehensive review. Constr. Build. Mater..

[138] Rana A.K., Rana S.B., Kumari A., Kiran V. (2009). Significance of nanotechnology in construction engineering. Int. J. Recent Trends Eng..

[139] Mallakpour S., Hussain C.M., Ajith S., Arumugaprabu V. (2021). Environmental and Occupational Health Hazards of Nanomaterials in Construction Sites. Handb. Consum. Nanoproducts.

[140] Onyango J. (2022). Health Impacts of Building Materials on Construction Workers. Ecological and Health Effects of Building Materials.

[141] Santhosh G., Nayaka G.P. (2022). Nanoparticles in Construction Industry and Their Toxicity. Ecological and Health Effects of Building Materials.

[142] Thakur M., Sharma A., Chandel M., Pathania D. (2022). Modern applications and current status of green nanotechnology in environmental industry. Green Functionalized Nanomaterials for Environmental Applications.

[143] Karnena M.K., Konni M., Saritha V. (2022). Occupational Health Problems of Construction Workers. Ecological and Health Effects of Building Materials.

[144] Gupta A.D., Patil S.Z. (2022). Potential Environmental Impacts of Nanoparticles Used in Construction Industry. Ecological and Health Effects of Building Materials.

[145] Joglekar S., Gajaralwar R. (2021). Potential risk and safety concerns of industrial nanomaterials in environmental management. Handbook of Nanomaterials for Wastewater Treatment.

[146] Cook L., Wantenaar C., Wise B. (2021). An Exploratory Study of the Cutting-Edge Development of Nanotechnology Pertaining to the Construction Industry. Mater. Sci. Forum.

[147] Singh D., Marrocco A., Wohlleben W., Park H.R., Diwadkar A.R., Himes B.E., Lu Q., Christiani D.C., Demokritou P. (2022). Release of particulate matter from nano-enabled building materials (NEBMs) across their lifecycle: Potential occupational health and safety implications. J. Hazard. Mater..

[148] Augustyniak A., Jablonska J., Cendrowski K., Głowacka A., Stephan D., Mijowska E., Sikora P. (2021). Investigating the release of ZnO nanoparticles from cement mortars on microbiological models. Appl. Nanosci..

[149] Allujami H.M., Jassam T.M., Al-Mansob R.A. (2021). Nanomaterials characteristics and current utilization status in rigid pavements: Mechanical features and Sustainability. A review. Mater. Today Proc..

[150] Valente M., Sambucci M., Sibai A. (2021). Geopolymers vs. Cement Matrix Materials: How Nanofiller Can Help a Sustainability Approach for Smart Construction Applications—A Review. Nanomaterials.

[151] García-Quintero A., Palencia M. (2021). A critical analysis of environmental sustainability metrics applied to green synthesis of nanomaterials and the assessment of environmental risks associated with the nanotechnology. Sci. Total Environ..

[152] Natarajan L., Jenifer M.A., Mukherjee A. (2020). Eco-Corona Formation on the Nanomaterials in the aquatic systems lessens their toxic impact: A comprehensive review. Environ. Res..

[153] Jin M., Li N., Sheng W., Ji X., Liang X., Kong B., Yin P., Li Y., Zhang X., Liu K. (2020). Toxicity of different zinc oxide nanomaterials and dose-dependent onset and development of Parkinson’s disease-like symptoms induced by zinc oxide nanorods. Environ. Int..

[154] Wu D., Ma Y., Cao Y., Zhang T. (2020). Mitochondrial toxicity of nanomaterials. Sci. Total Environ..

[155] Samadi S., Lajayer B.A., Moghiseh E., Rodríguez-Couto S. (2020). Effect of carbon nanomaterials on cell toxicity, biomass production, nutritional and active compound accumulation in plants. Environ. Technol. Innov..

[156] Caixeta M.B., Araújo P.S., Gonçalves B.B., Silva L.D., Grano-Maldonado M.I., Rocha T.L. (2020). Toxicity of engineered nanomaterials to aquatic and land snails: A scientometric and systematic review. Chemosphere.

[157] Rana A., Kalla P., Verma H.K., Mohnot J.K. (2016). Recycling of dimensional stone waste in concrete: A review. J. Clean. Prod..

[158] Ji Z., Guo W., Sakkiah S., Liu J., Patterson T.A., Hong H. (2021). Nanomaterial Databases: Data Sources for Promoting Design and Risk Assessment of Nanomaterials. Nanomaterials.

[159] Radnik J., Kersting R., Hagenhoff B., Bennet F., Ciornii D., Nymark P., Grafstrom R., Hodoroaba V.D. (2021). Reliable Surface Analysis Data of Nanomaterials in Support of Risk Assessment Based on Minimum Information Requirements. Nanomaterials.

[160] Ha J. (2021). Novel Applications of Nanoparticles in Nature and Building Materials. Novel Nanomaterials.

[161] Vera-Agullo J., Chozas-Ligero V., Portillo-Rico D., García-Casas M.J., Gutiérrez-Martínez A., Mieres-Royo J.M., Grávalos-Moreno J. (2009). Mortar and concrete reinforced with nanomaterials. Nanotechnol. Constr..

[162] He X., Shi X. (2008). Chloride permeability and microstructure of Portland cement mortars incorporating nanomaterials. Transp. Res. Rec..

[163] Karthick S., Park D.J., Lee Y.S., Saraswathy V., Lee H.S., Jang H.O., Choi H.J. (2018). Development of water-repellent cement mortar using silane enriched with nanomaterials. Prog. Org. Coat..

[164] Lee J., Mahendra S., Alvarez P.J. (2010). Nanomaterials in the construction industry: A review of their applications and environmental health and safety considerations. ACS Nano.

[165] Jones W., Gibb A., Goodier C., Bust P., Jin J., Song M. (2015). Nanomaterials in construction and demolition-how can we assess the risk if we don’t know where they are?. J. Phys. Conf. Ser..

[166] Al-Bayati A.J., Al-Zubaidi H.A. (2018). Inventory of Nanomaterials in Construction Products for Safety and Health. J. Constr. Eng. Manag..

[167] Jones W., Gibb A., Goodier C., Bust P., Song M., Jin J. (2019). Nanomaterials in construction—What is being used, and where?. Proc. Inst. Civ. Eng. Constr. Mater..

[168] Kamali S., Sanajou S., Tazehzadeh M.N. (2019). Nanomaterials in Construction and their Potential Impacts on Human Health and the Environment. Environ. Eng. Manag. J. (EEMJ).

[169] Lee J., Mahendra S., Alvarez P.J.J. (2009). Potential environmental and human health impacts of nanomaterials used in the construction industry. Nanotechnol. Constr..

[170] Sofranko A., Wahle T., Heusinkveld H.J., Stahlmecke B., Dronov M., Pijnenburg D., Hilhorst R., Lamann K., Albrecht C., Schins R.P. (2021). Evaluation of the neurotoxic effects of engineered nanomaterials in C57BL/6J mice in 28-day oral exposure studies. NeuroToxicology.

[171] Ndika J., Karisola P., Kinaret P., Ilves M., Alenius H. (2021). Profiling Non-Coding RNA Changes Associated with 16 Different Engineered Nanomaterials in a Mouse Airway Exposure Model. Cells.

[172] Wils R.S., Jacobsen N.R., Di Ianni E., Roursgaard M., Møller P. (2021). Reactive oxygen species production, genotoxicity and telomere length in FE1-Muta™ Mouse lung epithelial cells exposed to carbon nanotubes. Nanotoxicology.

[173] Adedara I.A., Anao O.O., Forcados G.E., Awogbindin I.O., Agbowo A., Ola-Davies O.E., Patlolla A.K., Tchounwou P.B., Farombi E.O. (2018). Low doses of multi-walled carbon nanotubes elicit hepatotoxicity in rats with markers of oxidative stress and induction of pro-inflammatory cytokines. Biochem. Biophys. Res. Commun..

[174] Migliore L., Uboldi C., Di Bucchianico S., Coppedè F. (2015). Nanomaterials and Neurodegeneration. Environ. Mol. Mutagenesis.

[175] Grande F., Tucci P. (2016). Titanium dioxide nanoparticles: A risk for human health?. Mini Rev. Med. Chem..

[176] Wiemann M., Vennemann A., Blaske F., Sperling M., Karst U. (2017). Silver nanoparticles in the lung: Toxic effects and focal accumulation of silver in remote organs. Nanomaterials.

[177] Gaharwar U.S., Meena R., Rajamani P. (2017). Iron oxide nanoparticles induced cytotoxicity, oxidative stress and DNA damage in lymphocytes. J. Appl. Toxicol..

[178] Ickrath P., Wagner M., Scherzad A., Gehrke T., Burghartz M., Hagen R., Radeloff K., Kleinsasser N., Hackenberg S. (2017). Time-dependent toxic and genotoxic effects of zinc oxide nanoparticles after long-term and repetitive exposure to human mesenchymal stem cells. Int. J. Environ. Res. Public Health.

[179] Hansen S.F., Hjorth R., Skjolding L.M., Bowman D.M., Maynard A., Baun A. (2017). A critical analysis of the environmental dossiers from the OECD sponsorship programme for the testing of manufactured nanomaterials. Environ. Sci. Nano.

[180] NIOSH (2011). Current Intelligence Bulletin 63, Occupational Exposure to Titanium Dioxide.

[181] NIOSH (2010). NIOSH Current Intelligence Bulletin Occupational Exposure to Carbon Nanotubes and Nanofibers.

[182] Raftis J.B., Miller M.R. (2019). Nanoparticle translocation and multi-organ toxicity: A particularly small problem. Nano Today.

[183] Spitzmiller M., Mahendra S., Damoiseaux R. (2013). Safety issues relating to nanomaterials for construction applications. Nanotechnology in Eco-Efficient Construction.

[184] Díaz-Soler B.M., Martínez-Aires M.D., López-Alonso M. (2016). Emerging risk in the construction industry: Recommendations for managing exposure to nanomaterials. Dyna.

[185] Yin Y., Yu S., Shen M., Liu J., Jiang G. (2015). Fate and transport of silver nanoparticles in the environment. Silver Nanoparticles in the Environment.

[186] Part F., Berge N., Baran P., Stringfellow A., Sun W., Bartelt-Hunt S., Mitrano D., Li L., Hennebert P., Quicker P. (2018). A review of the fate of engineered nanomaterials in municipal solid waste streams. Waste Manag..

[187] Holder A.L., Vejerano E.P., Zhou X., Marr L.C. (2013). Nanomaterial disposal by incineration. Environ. Sci. Process. Impacts.

[188] Liu Y., Zhu X. (2014). Measurement of formaldehyde and VOCs emissions from wood-based panels with nanomaterial-added melamine-impregnated paper. Constr. Build. Mater..

[189] Zheng S., Zhou Q., Chen C., Yang F., Cai Z., Li D., Geng Q., Feng Y., Wang H. (2019). Role of extracellular polymeric substances on the behavior and toxicity of silver nanoparticles and ions to green algae Chlorella vulgaris. Sci. Total Environ..

[190] Oh D., Noguchi T., Kitagaki R., Choi H. (2021). Proposal of demolished concrete recycling system based on performance evaluation of inorganic building materials manufactured from waste concrete powder. Renew. Sustain. Energy Rev..

[191] Tsydenova N., Becker T., Walther G. (2021). Optimised design of concrete recycling networks: The case of North Rhine-Westphalia. Waste Manag..

[192] Bonoli A., Zanni S., Serrano-Bernardo F. (2021). Sustainability in Building and Construction within the Framework of Circular Cities and European New Green Deal. The Contribution of Concrete Recycling. Sustainability.

[193] Gao C., Huang L., Yan L., Kasal B., Li W., Jin R., Wang Y., Li Y., Deng P. (2021). Compressive performance of fiber reinforced polymer encased recycled concrete with nanoparticles. J. Mater. Res. Technol..

[194] Dong Q., Wang G., Chen X., Tan J., Gu X. (2021). Recycling of steel slag aggregate in portland cement concrete: An overview. J. Clean. Prod..

[195] Guo F., Li H. (2021). Influence of Nanomaterials on Physical Mechanics and Durability of Concrete Composite Piers. Integr. Ferroelectr..

[196] Li W., Luo Z., Long C., Wu C., Duan W.H., Shah S.P. (2016). Effects of nanoparticle on the dynamic behaviors of recycled aggregate concrete under impact loading. Mater. Des..

[197] Agarwal A., Bhusnur S., Priya T.S. (2020). Experimental Investigation on Recycled Aggregate with Laboratory Concrete Waste and Nano-Silica. Mater. Today Proc..

[198] Hosseini P., Booshehrian A., Madari A. (2011). Developing concrete recycling strategies by utilization of nano-SiO 2 particles. Waste Biomass Valorization.

[199] Wang X., Cheng F., Wang Y., Zhang X., Niu H. (2020). Impact Properties of Recycled Aggregate Concrete with Nanosilica Modification. Adv. Civ. Eng..

[200] Mukharjee B.B., Barai S.V. (2015). Development of construction materials using nano-silica and aggregates recycled from construction and demolition waste. Waste Manag. Res..

[201] Zheng Y., Zhuo J., Zhang P. (2021). A review on durability of nano-SiO2 and basalt fiber modified recycled aggregate concrete. Constr. Build. Mater..

[202] Bang S.S., Lippert J.J., Yerra U., Mulukutla S., Ramakrishnan V. (2010). Microbial calcite, a bio-based smart nanomaterial in concrete remediation. Int. J. Smart Nano Mater..

[203] Barnat-Hunek D., Omiotek Z., Szafraniec M., Dzierżak R. (2021). An integrated texture analysis and machine learning approach for durability assessment of lightweight cement composites with hydrophobic coatings modified by nanocellulose. Measurement.

[204] Teizer J., Venugopal M., Teizer W., Felkl J. (2012). Nanotechnology and its impact on construction: Bridging the gap between researchers and industry professionals. J. Constr. Eng. Manag..

[205] Qureshi T.S., Panesar D.K. (2020). Nano reinforced cement paste composite with functionalized graphene and pristine graphene nanoplatelets. Compos. Part B Eng..

